# Evaluating the role of observational uncertainty in climate impact assessments: Temperature-driven yellow fever risk in South America

**DOI:** 10.1371/journal.pclm.0000601

**Published:** 2025-06-25

**Authors:** Sally Jahn, Keith Fraser, Katy A. M. Gaythorpe, Caroline M. Wainwright, Neil M. Ferguson

**Affiliations:** 1 MRC Centre for Global Infectious Disease Analysis, School of Public Health, Imperial College London, London, United Kingdom; 2 School of Earth and Environment, University of Leeds, Leeds, Yorkshire, United Kingdom

## Abstract

Global gridded temperature data sets (GGTDs) differ in data sources, quality control, generation methods, and spatial-temporal resolution, introducing observational uncertainty. This uncertainty is critical not only for studies on current climate conditions but also for future climate change projections, where observational data sets are used for bias correction and downscaling of global climate model (GCM) outputs. It is hence essential to ensure that reference data sets accurately represent the true climate state and span a sufficiently long period to filter out internal variability. The selection of appropriate GGTDs is hence a crucial yet often overlooked factor in research that examines the impact of climate variability and change on vector-borne diseases such as yellow fever (YF), a climate-sensitive arboviral disease endemic to tropical regions of Africa and South America. In this study, we evaluated four GGTDs, namely the Berkeley Earth Surface Temperatures (BEST), the Climatic Research Unit Time-Series (CRUTS), the fifth-generation atmospheric reanalysis of the global climate from the European Centre for Medium-Range Weather Forecasts (ECMWF), ERA5, and its land-focused derivative, ERA5Land, for health-related impact research, specifically examining YF transmission in South America. Each data set was evaluated via grid-based analysis and validated against national weather station data, focusing on Brazil and Colombia, where YF out-break risk remains. While reanalysis generally outperformed lower-resolution products, ERA5 demonstrated a slight advantage over ERA5Land despite the latter’s higher spatial resolution. Most importantly, our findings show that substantial differences among GGTDs affected the spatial representation of climate change indices, bioclimatic variables, and spatially aggregated temperature estimates at the administrative (AD) unit level, with substantial variations in the latter translating into markedly different estimates of key disease transmission parameters. In Colombia, admin-level temperature inputs differing by more than 6°C led to differences of about 0.2 in simulated reproduction numbers generated within a dynamic compartmental YF modeling framework.

## Introduction

Climate change has already aggravated the risk of various human pathogenic diseases, including vector-borne diseases (VBDs; please see S1 Glossary for a list of abbreviations and acronyms), and could further increase these risks in the future [1–3]. To effectively assess the impact of climate and, subsequently, future climate change on such diseases, it is essential to use high-quality observational data that are homogeneous, meaning they represent climate records that accurately reflect variations caused by climate processes and harmonized into a common format or standard. These data should also be continuous, have high spatial and temporal resolution, and have extensive temporal coverage. Global and regional gridded data sets are often used as alternatives to weather station data in disease-related climate impact studies. This is particularly prevalent for studies in low- and middle-income countries (LMICs) where ground-based networks typically face challenges such as sparse spatial and temporal coverage, limited documentation, inconsistent quality control, and restrictive data-sharing policies [4–7]. These gridded data sets, developed from diverse sources such as gauges, radar, satellite, and reanalysis, can help address these challenges but come with their own limitations. Each data set has specific strengths and weaknesses depending on the main applications for which it is designed, and all are susceptible to errors and biases, leading to observational uncertainty [8–10]. In climate impact modeling, observational data sets serve as inputs for data-driven models, and uncertainties in these data sources can significantly affect the outcomes of impact models across various fields, such as hydrological and epidemiological analysis, throughout the observation period [11–13]. Hence, the selection of observational data is suspected to also influence the outcomes of the infectious disease transmission model. Furthermore, the choice of observational data might strongly affect estimates of future simulated disease transmission, since uncertainties in observational data directly contribute to uncertainties in climate change projections [14–17]. This is because observational data sets serve as the reference climatology in climate change impact assessments, providing a baseline for assessing future changes and post-processing outputs from global climate models (GCMs) that are typically applied to model both current and future climate change effects.

GCMs are essential tools for understanding the climate system and projecting its evolution under different emissions scenarios. However, the output from GCMs remains coarse compared to the high-resolution data required for most impact studies, and systematic model errors introduce biases, observable as discrepancies between simulated and observed climate conditions. As a result, bias correction and downscaling (BC&D) are essential for applying GCM outputs to impact models. Downscaling methods can be broadly categorized into statistical and dynamical approaches. Statistical (bias correction) and downscaling techniques rely on hydrometeorological observations over a historical reference period to adjust model biases and refine the spatial resolution, ranging from simple methods accounting for changes in the mean of the quantity of interest (e.g., delta change methods) to more sophisticated approaches correcting biases in all quantiles of the distribution (e.g., quantile delta mapping) [18–20]. Dynamical downscaling involves using the output of a GCM as boundary conditions for a limited-area, higher-resolution regional climate model (RCM). RCM simulations are still prone to regional biases that need to be adjusted for climate change impact studies [19,21]. Consequently, any deficiencies in the reference based on the chosen observational data source are typically transferred to future climate change projections, regardless of the statistical or dynamical BC&D methods applied.

While several previous studies have projected an increase in transmission suitability for VBDs under future climate change [22–25], most have relied on simple statistical methods for the bias correction and downscaling of future climate change projections, such as linear scaling and delta change approaches [18,19]. These methods, which are straightforward, easy-to-implement, and widely adopted - for instance, by WorldClim [26,27] - primarily account for changes in mean values, effectively capturing broad climate change signals. However, they are too simplistic for modern climate impact studies as more comprehensive assessments, with the utilization of more advanced BC&D methods, are needed to consider changes across the full distribution of impact-driving climate variables, including extremes and shifts in seasonality. This is critical because these factors often result in the most significant consequences of climate change on VBDs and, therefore, are essential for research on future disease transmission and burden [28–30]. This emphasizes the importance of selecting observational data sets that accurately reflect the true state of the reference climate, including the distribution tails of impact-relevant climate variables, and highlights the need to address observational uncertainty in disease-related climate (change) impact modeling studies.

Therefore, observational data sets should be rigorously evaluated before being used as input data for disease transmission models to simulate and project disease transmission. This evaluation should ideally include comparative analyses, validation against weather station data, and sensitivity assessments of the impact models to the choice of data set. Moving beyond grid cell-based analysis is crucial, as epidemiological data are typically attributed to administrative (AD) units rather than regular grids. However, very few epidemiological studies have compared global gridded data sets with weather station data in the context of environmental epidemiology and climate-related health impact assessments [12,31] or have moved beyond grid-scale analyses to consider geographical units, with existing research focusing on high-income countries [32,33]. In this study, we assessed the utility and performance of widely used global gridded temperature data sets (GGTDs), representing viable reference data sets for bias correcting (and downscaling) climate model outputs in impact studies, and explored the sensitivity of AD-level VBD transmission risk to input data in South America. Our focus was on yellow fever (YF), a vaccine-preventable zoonotic arbovirus endemic to tropical regions of the continent. We used key disease parameter estimates based on a SEIR-type mechanistic dynamic model of yellow fever (YF) transmission [10], which is applied within the Vaccine Impact Modelling Consortium (VIMC) aiming to provide high-quality estimates of the public health impact of vaccination, to inform and improve policy and decision-making [34,35]. This model has previously been applied to evaluate the effectiveness of achieving vaccination coverage targets outlined in the World Health Organization’s Eliminate Yellow Fever Epidemics (EYE) Strategy [36], a key international framework for YF prevention and control. The model has also been adapted to account for the effects of weather and climate (change) on the dynamics of YF transmission [25]. We here specifically examined thermal conditions, as current and projected temperature has been identified as a key driver of YF transmission intensity [25], and because most previous evaluations of gridded data sets have primarily focused on precipitation [11,37,38]. We included an evaluation of the accuracy of GGTDs through comparisons with ground-based weather stations in Brazil (BRA) and Colombia (COL) in our analysis. These two countries not only contain AD areas with substantial differences in size, population distribution, orography, and climate, but are also at high risk for YF, with Brazil having experienced one of the most pronounced YF epidemics in recent years [39], with many YF cases emerging despite high vaccination coverage in some areas. The significance of yellow fever (YF) risk assessment has been underscored by recent epidemiological developments in 2025, with outbreaks resulting in 85 deaths (as of 16 May) and confirmed cases across five countries in the Region of the Americas, including Brazil and Colombia, prompting coordinated response efforts supported by the WHO [40].

Our main contributions were twofold: (1) comprehensively compare and evaluate global gridded temperature data sets with time series long enough to define baseline climate conditions, and (2) assess how sensitive simulated yellow fever (YF) reproduction numbers are to variations in temperature inputs at the first administrative level (AD1) by focusing on heterogeneous regions in Brazil and Colombia. Overall, our findings can inform recommendations on the utility of GGTDs, accounting for the regions of interest and their physiographic characteristics, and offer guidance for future studies assessing the impact of climate change on VBD transmission. Future research building on our work can enhance the evidence base needed to inform the design and timing of yellow fever interventions like vaccination strategies under evolving climatic conditions.

## Materials and methods

### Study domain

We focused our work on the South American domain between 40°S and 15°N, as this domain is particularly relevant for VBD research, encompassing all 13 main countries with endemic YF zones, where the disease remains a significant public health concern and all countries are considered to be at high risk [36]. These countries include the Pluri-national State of Bolivia, Brazil, Colombia, Ecuador, French Guiana, Guyana, Panama, Peru, Suriname, Trinidad and Tobago, and the Bolivarian Republic of Venezuela. Specifically, both Colombia and Brazil have reported YF cases in recent years and remain at risk for outbreaks [39,40].

**Fig 1:**
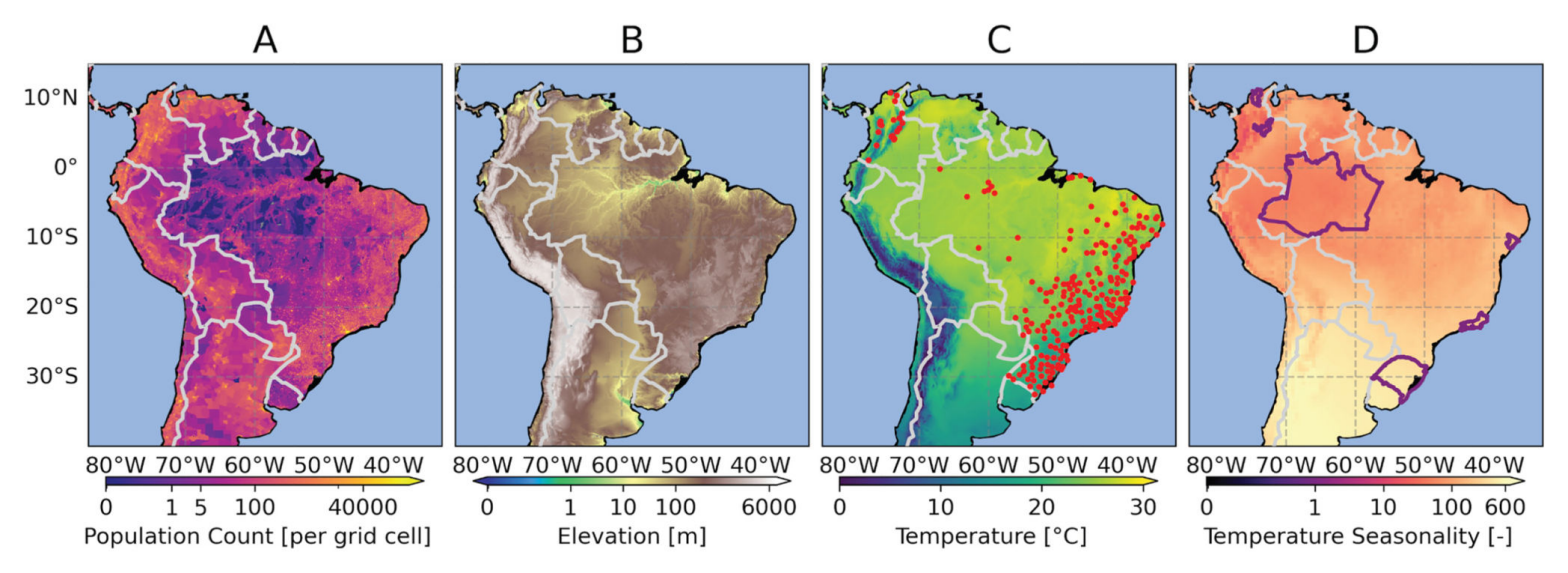
The South American study domain. A: Population distribution in 2010 [inhabitants per grid cell based on a 2.5 arc-minute resolution] [41]. B: Elevation [meters] [42]. C: Multi-year mean monthly temperature based on ERA5Land [°Celsius] [43]. D: Temperature seasonality [-] based on bioclimatic variable 4 (BCV4). Panels C and D refer to for the base period (1991-2020) and additionally show the locations of all selected weather stations (totaling 216 in Brazil and 20 in Colombia), collected form the Brazilian National Institute of Meteorology (INMET, [44]) and the Colombian Institute of Hydrology, Meteorology, and Environmental Studies (IDEAM, contacto@ideam.gov.co), and ValAr-P (4 in Brazil and 2 in Colombia), respectively. The boundaries of all areas are based on the Database of Global Administrative Areas, GADM (version 4.1) [45]. All maps display values across the chosen study domain in South America (north of 40° South).

Fig 1 illustrates the selected study domain in South America with respective country borders (in gray) and presents the geographical distribution of the population in 2010 (A), elevation (B) as well as the multi-year monthly mean temperature (C), and temperature seasonality (BCV4, D) derived from ERA5Land over the base period across South America (for further details please refer to Results). Panels C and D also highlight the locations of the weather stations (C) and six selected administrative (AD1) areas (D), marked by red dots and purple boundaries, respectively. The boundaries for Brazil and Colombia, used for country level and first level administrative (AD1) areas, were extracted from the Global Administrative Area Database (GADM), version 4.1 [45]. To illustrate the geographical distribution of population and elevation across both countries and South America, we used the Gridded Population of the World, Version 4 (GPWv4, Revision 11) for the year 2010 at a resolution of 2.5 arcminutes (approximately 5 km) [41] and acquired elevation data in meters (m) with a spatial resolution of 0.1° using the R package *elevatr* [46].

### Global gridded temperature data sets

We first present GGTDs, highlighting their diverse sources, properties, and spatial aggregation. Additionally, we introduce temperature extreme indices and bioclimatic variables (BCVs), which are widely used in climate change and species distribution modeling studies.

#### Data sources and aggregation

Table 1 provides a summary of the main characteristics of the selected observational GGTDs. We selected global information from reanalysis data sets, specifically ERA5 [47] and ERA5Land [43], as well as two additional gridded global data sets for air temperature, namely the Climatic Research Unit Time-Series (CRUTS) (version 4.07) [48,49] and Berkeley Earth Surface Temperatures (BEST) data sets [50,51]. These products were chosen because they represent three (excluding ERA5Land) of the five main global data sets for air temperature in the Intergovernmental Panel on Climate Change (IPCC) Atlas [52]. The IPCC Atlas emphasizes the importance of assessing observational uncertainty when evaluating and attributing historical trends, and it highlights that climate change impact assessments should rely on integrated analyses from multiple data sets. We chose not to include either W5E5 [53], which is based on the bias-adjusted ERA5 reanalysis over land (WATCH Forcing Data applied to ERA5, WFDE5), or the Hadley Centre Climate Research Unit version 5 data set (HadCRUT5) from the Climate Research Unit of the University of East Anglia [54]. This decision was made because we aimed to focus on data sets that are as independent as possible and derived from different sources. However, ERA5Land from the European Centre for Medium-Range Weather Forecasts, which covers only land surfaces and is an enhanced reprocessing of the land component using a higher resolution model version, with ERA5 as an input, was included to evaluate the impact of changing spatial resolution in reanalysis. Similarly, we harmonized and re-gridded the original data sets to match a medium resolution of 0.5°. This approach was implemented to avoid unfairly penalizing low-resolution data sets and, more importantly, to systematically account for the impact of spatial resolution changes in our analysis of observational uncertainty. All chosen data sets are regularly updated, well-established, and widely used in climate and environmental sciences.

We temporally aggregated hourly temperature observations for reanalysis data and calculated daily mean temperatures for all grid cells across the South American domain. Next, we temporally aggregated gridded daily observations from BEST, ERA5Land, and ERA5 to obtain gridded monthly averages. Additionally, we extracted or generated gridded daily minimum and maximum temperatures (TN and TX) for BEST and reanalysis, respectively. We spatially aggregated and estimated mean values across the grid-specific daily mean temperatures of grid cells within or intersecting the boundaries of the corresponding AD1 areas in Brazil and Colombia. As these area-level temperature averages were calculated using data from a regular latitude-longitude grid, we applied latitude-based weights (using the cosine of latitude) to account for meridian convergence at higher latitudes. Overall, we produced eight daily temperature timeseries for each AD1 area, corresponding to each selected GGTDs, available both at their native resolutions and on a common 0.5° grid. We subsequently also temporally aggregated the daily area-level temperature timeseries (BEST and reanalysis) into monthly timeseries data, which serves as the primary temporal focus of our analysis. For GGTD-only analyses, we defined a 30-year base period spanning from 1991 to 2020.

#### Extreme indices and bioclimatic variables

We used established extreme indices to assess how uncertainties in GGTDs affect the derivation and detection of temperature extreme events, such as the frequency of summer days or tropical nights. These indices were generated based on daily minimum and maximum temperature data (excluding CRUTS), following the definitions provided by the Expert Team on Climate Change Detection and Indices (ETCCDI) [55,56]. Additionally, we used a selection of bioclimatic variables, calculated from climatological monthly means of temperature, which are physiologically important and have been frequently used for species distribution modeling and in VBD research [57,58]. Please refer to Table 2 for further details. We used the Climate Data Operators (CDO) software [59] to derive ETCCDI and the function BIOVARS from the R package *dismo* to produce BCV estimates.

### Weather station data

Temperature data at daily resolution were obtained from the Brazilian National Institute of Meteorology (INMET, available online at [44]). The Colombian Institute of Hydrology, Meteorology, and Environmental Studies (IDEAM) originally reported and provided temperature data at (sub-)hourly resolution (contacto@ideam.gov.co). Data for Brazil were directly downloaded from publicly available sources via the official INMET website, with documentation provided in English. In contrast, data for Colombia were obtained through direct communication with IDEAM. The Brazilian data set included a greater number of stations, consistently reporting both temperature and precipitation, with records already aggregated at a daily temporal scale. In Colombia, fewer stations were provided, and the records were available at sub-daily scales. The availability of temperature and precipitation data was not consistent across Colombian stations, with some only providing information for one of the two variables. We standardized the data for both countries into a uniform format, removing unreliable measurements based on extreme outliers (e.g., daily values above 55.00°C), and verifying date availability and completeness. For certain stations, especially those along the coast, some GGTDs, in particularly ERA5Land, did not provide grid-based information at native resolutions, leading to the exclusion of stations to address discrepancies between station locations and grid points. To construct reliable daily timeseries for Colombia, we aggregated (sub-)hourly data into daily values, requiring a minimum of 70% data availability per day. To reduce the impact of missing values, we selected only stations with over 70% daily coverage per month during the evaluation period from 2011 to 2020 for both Brazil and Colombia. This evaluation period was chosen to ensure consistent and adequate data coverage across the study domain, and only stations with data available for each year within this period were included in the analysis.

### Yellow fever data and model

We utilized a previously described dynamic model of YF transmission estimated from relevant epidemiological data sources [10]. The model is a dynamic compartmental model of YF transmission in humans assuming both a risk of infection through spillover (parametrized by the spillover force of infection or FOI) and a risk of infection through the ‘urban’ cycle characterized through a human-human reproduction number, mediated by mosquitoes. These epidemiological parameters are assumed to depend on environmental covariates such as a) Ae. aegypti occurrence, b) middle infrared reflectance, c) non-human primate species richness, d) Human population size, e) Temperature suitability index (developed from Gaythorpe et al. 2021 [25]), and f) the type of land cover. To estimate reproduction number, Fraser et al. used available seroprevalence, case notification and death notification data within a Bayesian framework. This characterized the relationship between epidemiological parameters and environmental inputs.

In order to project time-varying values of reproduction number for this analysis, we replaced the time-invariant temperature suitability index in the original analysis with time-varying values calculated from temperature data derived from each GGTD (see next section, specifically Temperature-yellow fever associations, for details).

### Validation areas

A GADM AD1 unit was designated as a validation area (ValAr) if it contained at least one selected weather station. The evaluation of GGTDs, comparison of aggregated area-level estimates, and assessment of YF human-to-human transmission were conducted in selected ValAr in Brazil and Colombia, referred to as primary validation areas (ValAr-P). These ValAr-P were selected based on specific criteria. We prioritized regions with available weather station data that represented diverse climatic zones and landscapes. Additionally, the ValAr exhibited a range of behaviors in terms of seasonality, observational data set uncertainty, and reproduction number. The ValAr-P included regions where the estimated reproduction number is consistently higher than 1.00 (giving the potential for self-sustaining outbreaks), regions where the estimated reproduction number is consistently below 1.00, and regions where the estimated reproduction number fluctuates above and below 1.00 depending on time of year, data source, and/or parameter set.

### Evaluation methods and metrics

The most common approach to compare ground-based observations with gridded data products, such as satellite-based estimates and climate model outputs, is a station-to-grid cell comparison. Accordingly, we compared individual station timeseries with corresponding grid cell values by applying three standard statistical methods, namely the Pearson correlation coefficient (PCC), the mean absolute error (MAE), and the root mean square error (RMSE). PCC was reported only when statistically significant at the 95% confidence level. Additional details on the metrics can be found in S1 Methods in the Supplement.

We assessed the overall performance and accuracy of each product across all ValAr by taking the mean of the results of multiple individual station-to-grid cell comparisons within each ValAr, providing an evaluation of how well each product performed across the entire area during the evaluation period. Additionally, we evaluated the monthly area-specific temperature distributions and the climatological annual temperature cycles derived from the aggregated area-level timeseries for each GGTD in each ValAr-P. This GGTD-only analysis allowed us to evaluate more in-depth data set biases, accounting for underlying climatological differences, and to determine whether these biases followed a specific seasonal pattern or remained relatively constant throughout the year over the base period.

To assess the effect of different GGTDs on simulated YF transmission intensity, we used the existing model for estimating YF reproduction number from environmental covariates [10]. The time-varying (daily or monthly depending on data set) temperature values from the GGTDs were substituted for the original mean temperature data set used when the model parameters were estimated. These temperature values were used to calculate new values of temperature suitability [25], from which time-varying values of YF reproduction number were calculated using the model. Monthly values of reproduction number based on each GGTD were then compared.

## Results

### Observational data uncertainty

We present the results of our analysis of grid-based observational uncertainty. This included assessing the variation among GGTDs in terms of the geographical distribution of temperature and the derived ETCCDI and BCVs across the South American study domain. Given that ERA5Land is often used in health-related climate impact assessments [12,60] due to its enhanced spatial resolution for meteorological variables, it is used here as a reference when comparing the different GGTDs. The validation of GGTDs was conducted through a comparison of grid cell and station-based temperature timeseries in Brazil and Colombia.

#### Study setting and differences in GGTDs

Originally, INMET’s network included data from over 600 meteorological stations, from which 216 were selected based on predefined criteria and an evaluation period starting in 2011. In Colombia, although more than 340 records were available through IDEAM, only 20 stations met all selection criteria during the evaluation period and were included in the analysis. Fig 1 shows the locations of the in total 216 selected weather stations in Brazil and 20 in Colombia, corresponding to 23 and 12 ValAr regions, respectively. The selected ValAr-P include Amazonas (BRA4), Rio de Janeiro (BRA19), Rio Grande do Sul (BRA21), and Sergipe (BRA26) in Brazil (BRA) as well as Boyacá (COL7) and Magdalena (COL20) in Colombia (COL), highlighted (in purple) in panel D. The figure reveals a clear pattern: weather stations are predominantly located in or near densely populated areas, resulting in a bias in the analysis toward regions with higher population densities in each country, which were also more relevant for our health-related context. In Brazil, for example, stations tend to cluster along coastal regions, representing areas of elevated population density. This pattern strongly influenced the selection of ValAr. The figure also high-lights the diverse environmental conditions across Brazil and Colombia, emphasizing the importance of considering regional diversity in environmental factors when selecting ValAr. The selected ValAr-P demonstrated considerable variation in both size and environmental characteristics, as shown, e.g., by temperature seasonality (panel D).

S1 Fig provides a more detailed overview of the selected weather stations and ValAr-P in Brazil and Colombia (A), also illustrating the variation in spatial resolution across all GGTDs (B), using the example of the Brazilian ValAr-P, Rio de Janeiro (BRA19). Furthermore, the clear influence of the land-sea mask on the selection of grid cells for BRA19 along the coast-line in ERA5Land becomes evident, with a similar impact expected in all coastal AD areas. Additionally, S1 Table in the Supplement provides an overview of all ValAr, including information on average population, area size, elevation, and the number of grid cells selected for spatial aggregation per GGTD for each ValAr, based on the respective gridded data sets. It also includes details on the number of weather stations within each ValAr and the respective temperature characteristics, derived from the timeseries data averaged across all stations in each area. Station altitudes varied between about 2m and 1663m (mean 515m) across Brazil and between 1m and 3510m (mean 1623m) across Colombia. In Colombia, the mean of the stations’ average monthly temperature ranged from 8.38°C in Cundinamarca (1 station), where temperatures varied between 3.01°C and 10.53°C, to 29.33°C in Cesar (2 stations), with temperatures ranging from 26.94°C to 31.62°C. In Brazil, the mean monthly temperature across stations was between 18.57°C in Rio Grande do Sul (26 stations), where the temperatures ranged from 11.35°C to 24.05°C, and 27.23°C in Piauí (3 stations), with temperatures varying from 24.47°C to 30.71°C.

#### Differences in ETCCDI and BCVs

**Fig 2:**
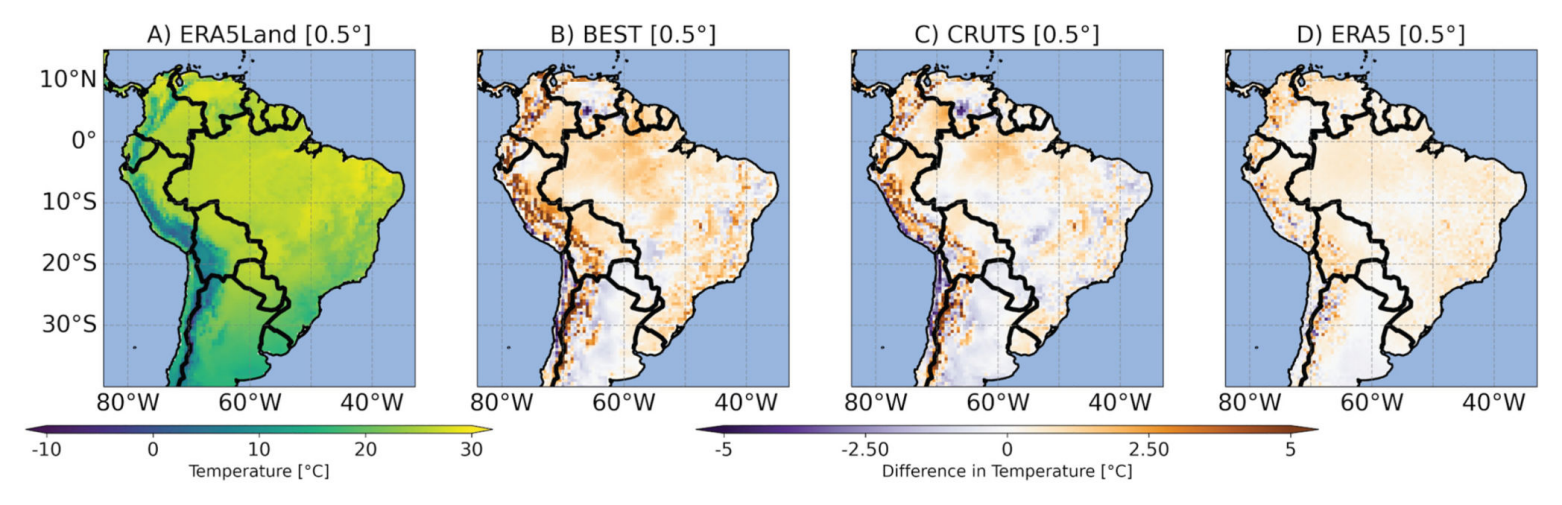
Bioclimatic variable 1 (BCV1). A: Mean annual temperature [°C] for ERA5Land. B-D: For all other global gridded temperature data sets (GGTDs), the difference [°C] compared to ERA5Land is shown. All values represent averages over the base period (1991-2020). The maps are presented on a common 0.5° grid.

Differences in GGTDs might impact not only the average temperature conditions but also the representation of spatial and temporal patterns and the calculation of extreme indices and bioclimatic variables. Fig 2 presents the climatological mean annual temperature (BCV1) derived from ERA5Land, alongside the differences between ERA5Land and all other GGTDs, averaged over the base period (1991-2020), across the South American study domain. To enable comparisons, the 0.5° resolution version of each data set was used. The figure reveals that GGTDs exhibited varying degrees of temperature differences across data sets and study domain, with pronounced discrepancies in coastal areas and regions with complex terrain, such as the Andes. Unsurprisingly, since ERA5 is the forcing input for ERA5Land, the analysis generally showed smaller temperature differences between these two model-based data sets. More specifically, BEST, and to a lesser extent CRUTS, showed a warm bias across large parts of the study domain when compared with ERA5Land, while colder climatological mean annual temperatures were observed in southern areas, particularly Argentina, with a similar but less pronounced pattern in ERA5; additionally, CRUTS displayed more patchy areas of colder temperatures in Brazil compared to the other data sets.

**Fig 3:**
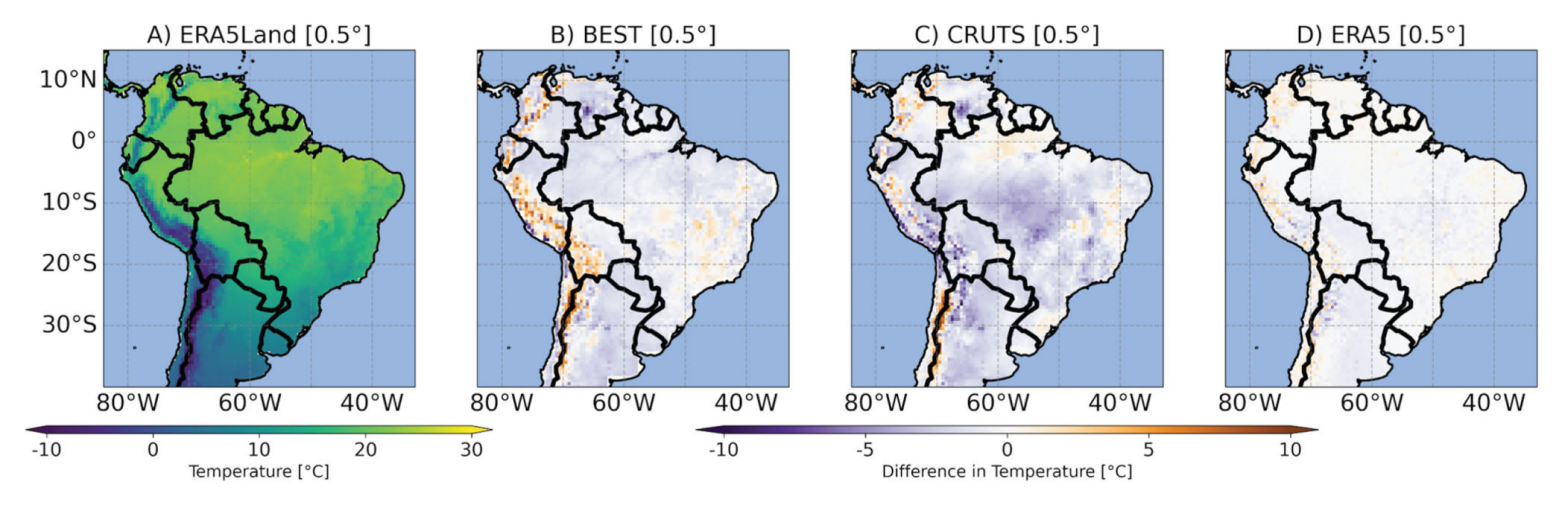
Bioclimatic variable 6 (BCV6). A: Minimum temperature of the coldest month [°C] for ERA5Land. B-D: For all other global gridded temperature data sets (GGTDs), the difference [°C] compared to ERA5Land is shown. All values represent averages over the base period (1991-2020). The maps are presented on a common 0.5° grid.

**Fig 4:**
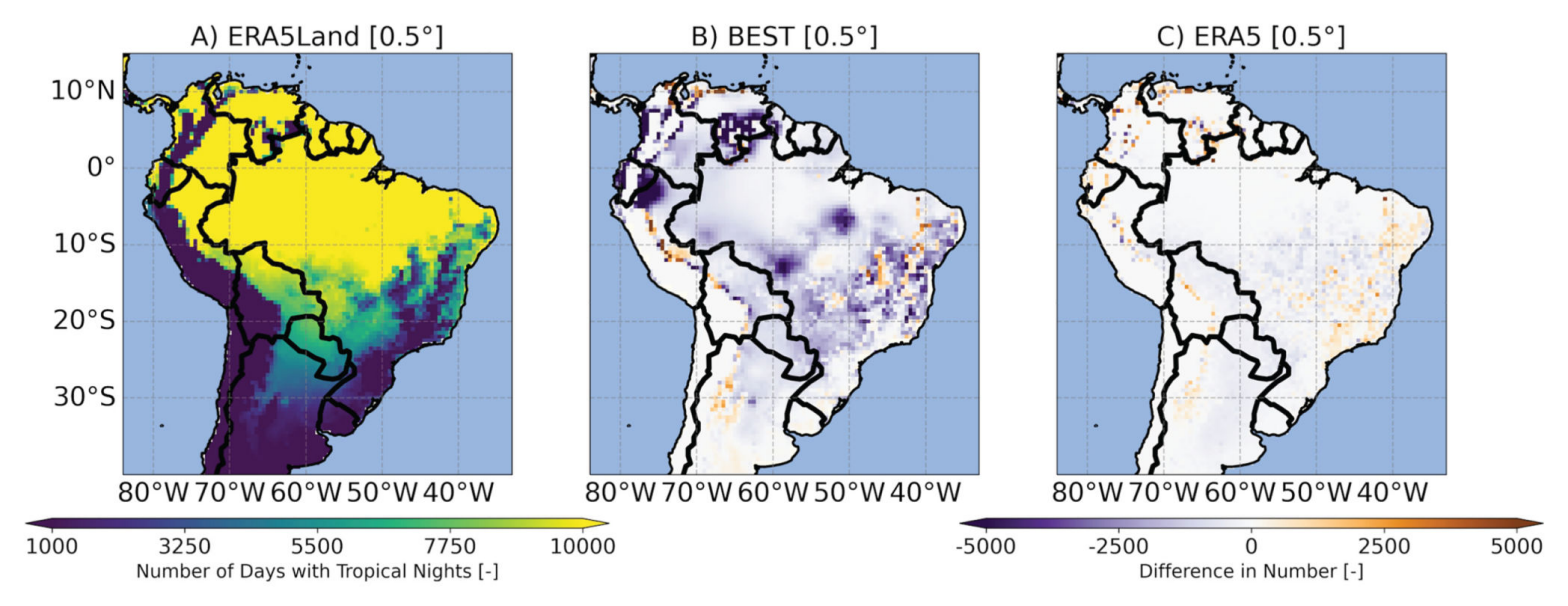
Tropical nights index per time period (TR). A: Number of tropical nights [-] for ERA5Land (TR). B-C: For all other global gridded temperature data sets (GGTDs), the difference in number [-] compared to ERA5Land is shown. All values represent averages over the base period (1991-2020). The maps are presented on a common 0.5° grid. Note that the Climatic Research Unit Time-Series data set (CRUTS) was excluded from the analysis, as TR was calculated using daily values.

Given that maximum and minimum temperatures (including night-time conditions) are particularly important for mosquito survival [61], Figs 3 and 4 exemplarily illustrate our findings for BCV6 (minimum temperature of the coldest month based on climatological monthly means) and TR (tropical nights based on TN). For BCV6, differences among GGTDs were more pronounced in BEST and CRUTS, with a warm bias when compared to ERA5Land evident in the Andes-dominated regions. CRUTS showed a pronounced cold bias over large parts of Brazil. Regarding tropical nights, differences in GGTDs in Fig 4 - especially in BEST - highlight a strong underestimation (and some very localized overestimation) of tropical nights during the base period. Inconsistencies across GGTDs affected the spatial representation of all other BCVs and ETCCDI indices, also including those focusing on maximum (daily) temperatures. For the visualization of respective results, see S2 Fig.

#### Comparison against weather station data

Table 3 presents the statistical evaluation of monthly temperature timeseries, showing averaged results from multiple station-to-grid cell comparisons within each ValAr-P. Additionally, the table provides means calculated across all 23 and 12 ValAR in Brazil and Colombia, respectively. Comparisons between station data and GGTDs across ValAr were conducted using data sets at their native resolution and on a common 0.5° grid (values in brackets).

The evaluation revealed varying levels of agreement, with timeseries from individual stations in Colombia, in general characterized by the influence of the Andes and a low degree of seasonality across the country, generally showing greater divergence from corresponding GGTD grid cells compared to those in Brazil. When considering individual ValAr, agreement between data sets showed relatively small variations in Brazil but varied noticeably in Colombia. Overall, across the ValAr-P, weaker agreement was observed in remote areas, such as Amazonas (BRA4) in Brazil, and in regions with complex topography, such as Boyacá (COL7) in Colombia, where stronger differences between data sets were evident. Seasonality appeared to have a lesser impact on the evaluation, as ValAr-P with greater temperature variability or more extreme seasonal changes did not tend to show larger differences in metrics.

On average, across all ValAr in both Colombia and Brazil, the mean values for all metrics indicated higher agreement between station data and grid cells in Brazil, with ERA5 emerging as the best-performing data set in both countries. Correlation values suggested a weaker alignment in trends and patterns of temperature between station and grid cell values, while RMSE and MAE metrics indicated more pronounced deviations of gridded data sets from observed measurements for Colombia when compared to Brazil.

The influence of spatial resolution with GGTDs having coarser native resolutions tending to show improved agreement when re-gridded to a finer grid was only strongly evident in Boyacá (COL7) across the ValAr-P, where GGTDs with finer native resolutions tended to exhibit a slightly weaker performance when evaluated on a common 0.5° grid. In contrast, the impact of re-gridding was minimal in the ValAr-P of Brazil and, on average, across all ValAr in both Brazil and Colombia, showing only minor changes in metrics.

**Fig 5:**
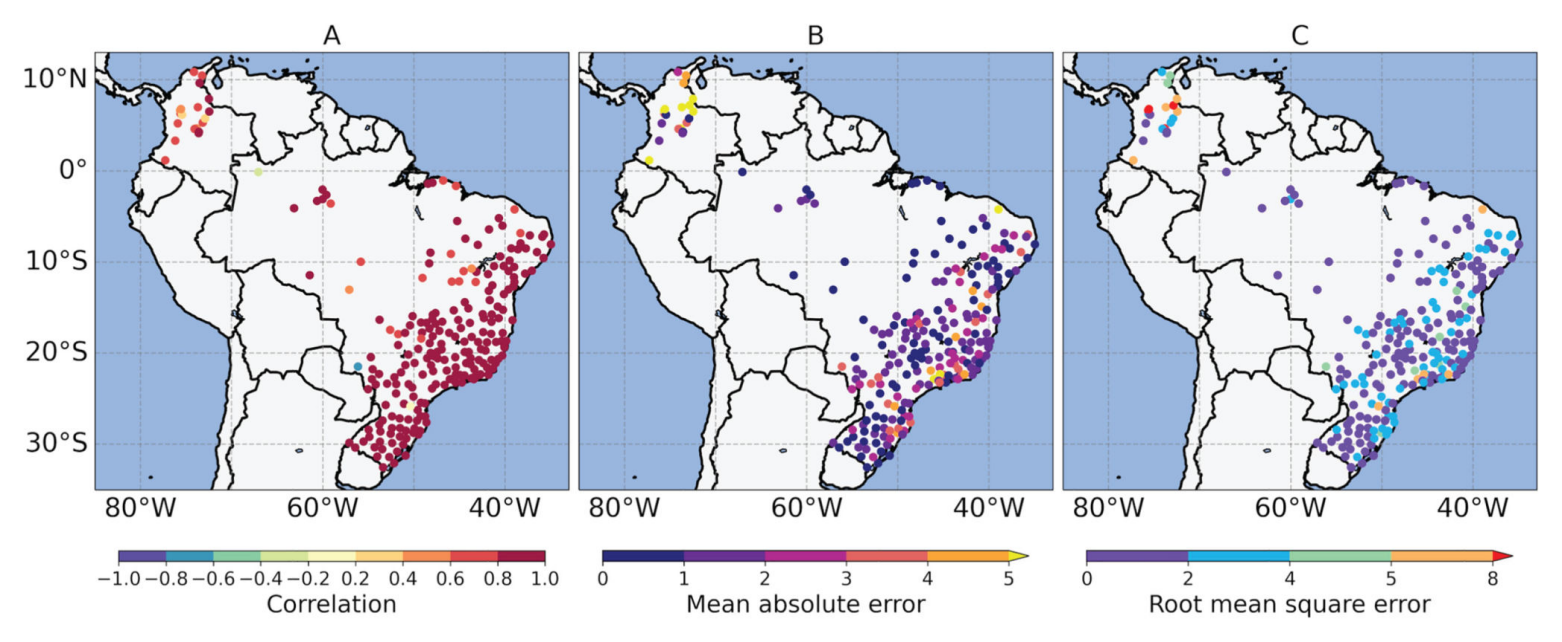
Spatial maps of evaluation metrics. Comparison of grid cell and station values based on A: Pearson correlation coefficient (PCC). B: Mean absolute error (MAE). C: Root mean square error (RMSE) between ERA5Land and ground-based observations at the corresponding station on a monthly time scale in Brazil and Colombia.

Fig 5 illustrates the spatial patterns of agreement between monthly station data and grid cell-level estimates across Brazil and Colombia for ERA5Land. As expected, weaker agreements between point and grid cell timeseries were more pronounced in Colombia, particularly in terms of MAE and RMSE. Additionally, a few locations in Brazil displayed relatively high MAE and RMSE values, while only a small number of locations exhibited low, and occasionally negative, correlations. However, no distinct spatial patterns were evident in either country.

### Area-level temperature estimates and YF transmission

We now describe how the four GGTDs translated into varying spatially aggregated temperature estimates and demonstrate how these deviations led to substantial variations in simulated area-level YF human-to-human transmission. Our analysis focused on ValAr-P.

#### Area-level temperature timeseries

**Fig 6:**
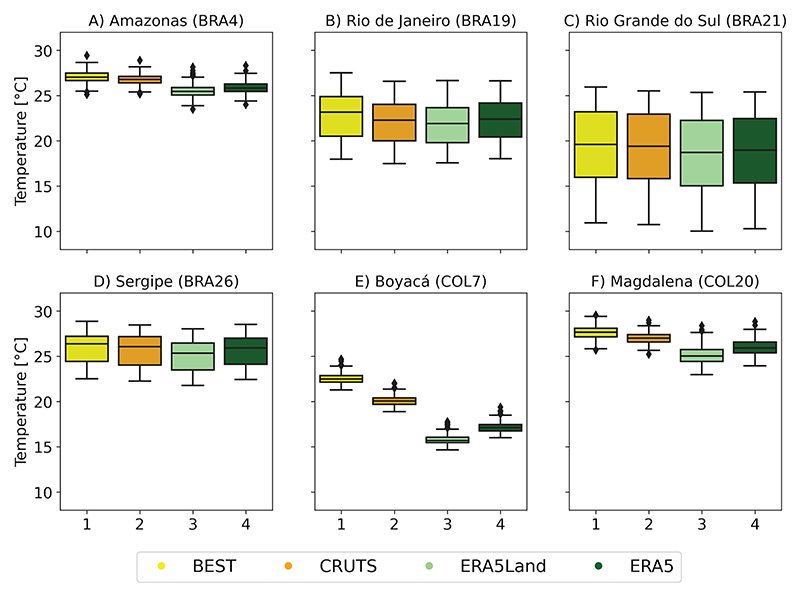
Boxplots comparing the temperature distributions of monthly timeseries based on different global gridded temperature data sets (GGTDs), averaged across each primary validation area (ValAr-P) in Brazil and Colombia, for the base period (1991-2020). The figures are organized as follows: A: Amazonas (BRA4) B: Rio de Janeiro (BRA19) C: Rio Grande do Sul (BRA21) D: Sergipe (BRA26) E: Boyacá (COL7) F: Magdalena (COL20) in Brazil (BRA) and Colombia (COL), respectively. These boxplots are based on area-level temperature timeseries derived from GGTDs at their native spatial resolution.

**Fig 7:**
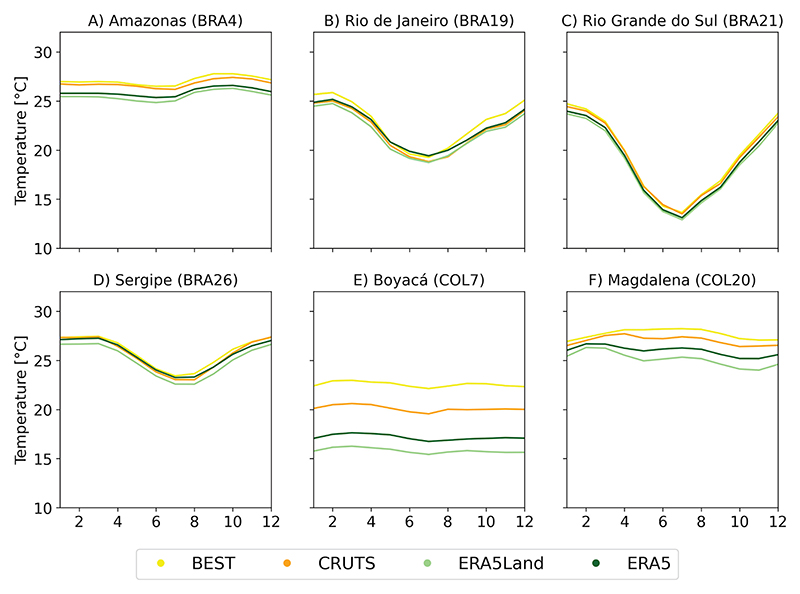
Climatological annual cycles of monthly temperature (°C) for the six primary validation areas (ValAr-P) selected across Brazil and Colombia, calculated over the base period (1991-2020). The figures are organized as follows: A: Amazonas (BRA4) B: Rio de Janeiro (BRA19) C: Rio Grande do Sul (BRA21) D: Sergipe (BRA26) E: Boyacá (COL7) F: Magdalena (COL20) in Brazil (BRA) and Colombia (COL), respectively. These climatological annual cycles are based on area-level temperature timeseries derived from global gridded temperature data sets (GGTDs) at their native spatial resolution.

For ValAR-P, we compared area-level temperature estimates from GGTDs at their native resolution, see Figs 6 and 7, with those interpolated to a common 0.5° grid, see S3 Fig. We assessed differences in aggregated timeseries by examining monthly area-specific temperature distributions and corresponding climatological annual cycles across the base period. These results should be interpreted within the context of each country’s specifics characteristics, including the varying sizes and locations of AD1 areas, as illustrated in Fig 1 and S1 Table.

As expected, the boxplots and annual cycles reveal minimal temperature fluctuations and seasonality for both Colombian ValAr-P, with a clear increase in temperature variability from northern to southern areas across all selected ValAr-P in Brazil. While this trend is evident in the rise of the interquartile range (IQR) shown in Fig 6, from the northernmost ValAr-P, the Amazon region (BRA4) with an IQR of 0.84°C for ERA5Land (BEST: 0.81, CRUTS: 0.71, ERA5: 0.82; in°C), to the southernmost area, Rio Grande do Sul (BRA21) with an IQR of 7.22°C for ERA5Land (BEST: 7.22, CRUTS: 7.12, ERA5: 7.13; in°C), it is particularly demonstrated by the seasonal patterns depicted in Fig 7. Overall, all data sets, when compared to ERA5Land, showed a tendency to shift towards warmer temperatures across all ValAr-P.

While area-level temperature timeseries for most ValAr-P areas in Brazil show strong agreement across data sets, significant discrepancies were observed in Colombia, particularly in the more mountainous region of Boyacá (COL7), and to a lesser extent in Magdalena (COL20). Mean temperature values varied in Boyacá (COL7) from ERA5Land (15.81°C) to BEST (22.56°C). In Brazil, aggregated temperature estimates were generally more consistent, although slight differences were observed in the remote Amazon region (BRA4), despite it being the largest Brazilian ValAr. Mean temperature values varied in BRA4 from ERA5Land (25.53°C) to BEST (27.08°C). Notably, Sergipe (BRA26), the smallest ValAr-P, exhibited minor differences between timeseries, mean temperature values varying from ERA5Land (25.05°C) to BEST (25.90°C). Thus, the spatial extent over which the GGTDs were aggregated was not a primary factor influencing the observed differences in GGTDs and hence the associated observational uncertainty. However, temperature estimates derived from GGTDs re-gridded to a common 0.5° grid showed more consistent and closely aligned timeseries, both in terms of monthly distributions and climatological annual cycles, in Boyacá (COL7). This area also exhibited the most pronounced differences in our station-based evaluation of GGTDs, with reanalysis showing the strongest agreement with station data (please refer to section titled Comparison against weather station data).

Differences across data sets remained relatively consistent throughout the year for most ValAr-P, with the strongest inconsistencies noted in Boyacá (COL7, Fig 6). Aggregated area-level temperature estimates were thus largely unaffected or only negligibly impacted by seasonality across the ValAr-P.

#### Temperature-yellow fever associations

**Fig 8:**
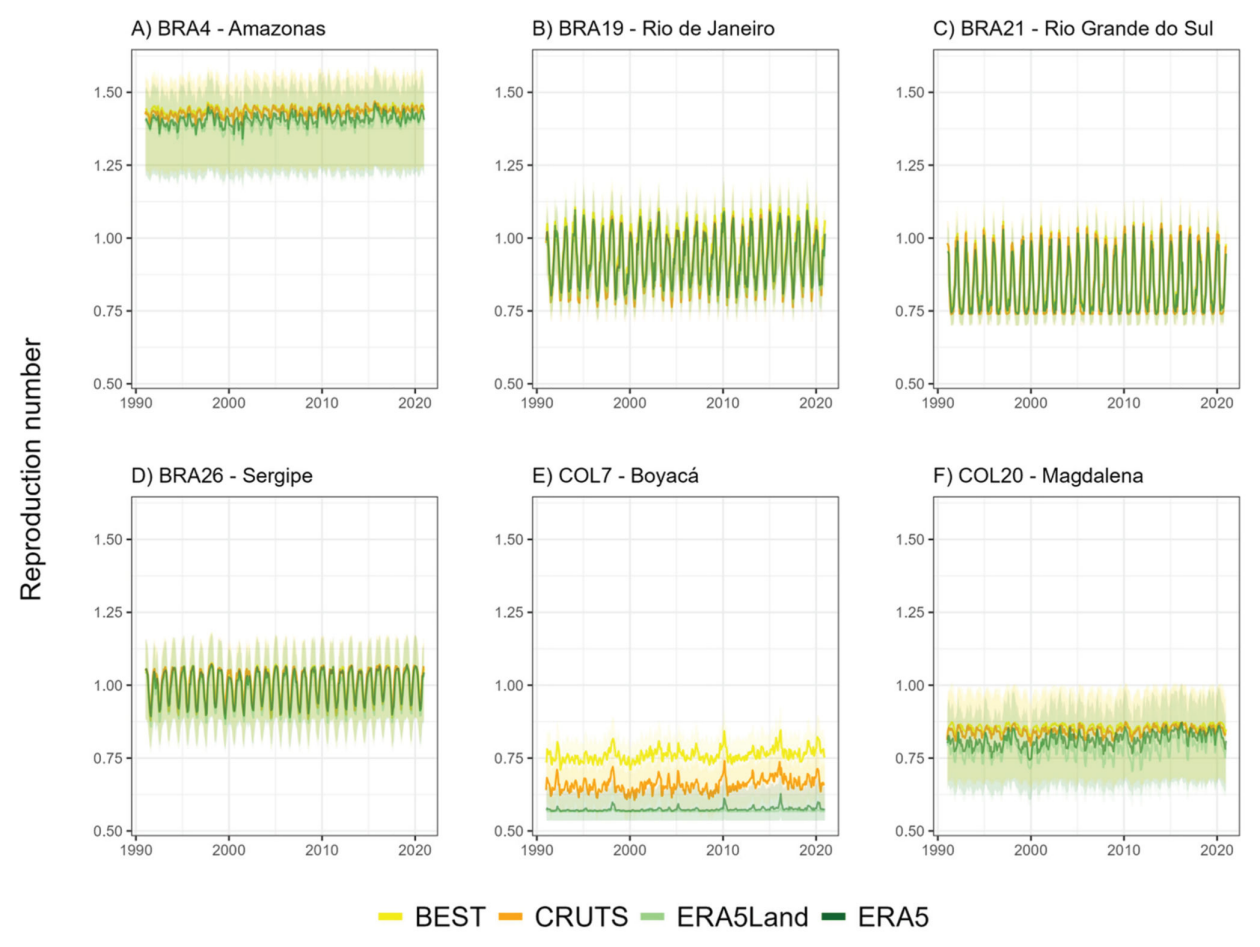
Calculated values of reproduction number (B) for the six primary validation areas (ValAr-P) selected across Brazil and Colombia, calculated over the base period (1991-2020). Displayed values are monthly averages calculated from daily values calculated from environmental covariates listed in Yellow fever data and model, with temperature suitability calculated from daily temperature values taken from the four GGTDs. For CRUTS, only monthly temperature values were available, so reproduction number values were calculated directly on a monthly basis.

Time-varying values of epidemiological parameters (reproduction number) were calculated based on time-varying temperature data shown in Fig 6. This was done by converting temperature to temperature suitability[25] and recalculating parameter values using the method described in [10]. The results are shown in Fig 8. The magnitude of seasonal variation in parameter values and the degree of difference between GGTDs varies between the selected regions.

The reproduction number threshold of 1.00 is critical, delineating the boundary between declining (reproduction number below 1.00) and sustained or expanding transmission (reproduction number above 1.00). The selected regions show different regimes in terms of how seasonal variation affects the calculated reproduction number. One region (Amazonas in Brazil) has the reproduction number consistently higher than 1.00, indicating that outbreaks can be sustained all year round. Two other regions (Magdalena and Boyacá in Colombia) have the reproduction number consistently below 1.00, indicating that outbreaks cannot be sustained at any time of the year. The remaining three regions show variation of the reproduction number between values below 1.00 and values close to or above 1.00 at different times of the year, indicating that the sustainability of outbreaks in these regions may vary seasonally.

The numerically largest difference in epidemiological parameter values between GGTDs is seen in Boyacá, due to that region having the largest temperature variation between data sets. However, this difference is not substantial due to the low temperatures in the region - the reproduction number is consistently the lowest among the selected regions and remains consistently below 1.00.

## Discussion

This study evaluated four GGTDs to serve as data inputs for impact models and as reference data sets for bias correcting (and downscaling) climate model outputs to assess the impacts of climate and climate change on YF transmission. We assessed whether differences in spatially aggregated temperature estimates derived from the different GGTDs translated into variations in the estimated YF reproduction number. Our analysis focused on Brazil and Colombia, not only due to their diversity in size, population density, orography, and temperature patterns, but also because both countries have a history of reported yellow fever cases.

### Grid-based differences and station-based validation of GGTDs

While the GGTD-only analysis showed no clear seasonal pattern in differences between data sets, similarly, seasonality did not strongly influence station-to-grid cell comparisons, as ValAr-P, despite varying temperature variability throughout the year, did not show a clear and consistent impact on differences in metrics. However, remote areas and regions with complex terrain in both countries showed lower agreement between GGTDs and station data, as well as between the data sets themselves. Spatial resolution proved crucial when validating GGTDs, especially in areas with complex terrain.

Our grid-based analysis of geographic distributions, extreme temperature indices, and bio-climatic variables revealed distinct regional variations in grid-based GGTD-estimated temperatures, with pronounced differences in mean temperature patterns observed in Andes-dominated and coastal areas. Regions with notable differences in ETCCDI and BCVs estimates often coincided with areas of pronounced differences between GGTDs in mean temperature patterns but also shifted or extended to other parts of South America. The observed variations in GGTDs, such as differences of over 5.00°C in diurnal and annual temperature ranges or in the number of tropical nights equivalent to over six years, could strongly impact the simulated distribution and occurrence of disease-transmitting vectors.

Validation of GGTDs against weather station data confirmed that temperature is generally more predictable than precipitation, as rainfall is well known to be highly variable due to its dependence on complex atmospheric dynamics, local topography, and short-term weather events. Hence, in contrast to rainfall, data set performance for temperature is more consistent and more dependent on the spatial resolution of a GGTD. For instance, higher-resolution GGTDs such as reanalysis, namely ERA5 and ERA5Land, consistently outperformed lower-resolution ones such as CRUTS and BEST. Spatial interpolation of BEST did not notably improve accuracy in most validated areas, as it failed to add meaningful spatial information to improve data set precision. Re-gridded reanalysis data sets, with their initial finer native spatial resolution and detail, showed minimal variations in metrics and consistently out-performed lower-resolution GGTDs when compared with CRUTS and BEST on a 0.5° grid. However, in regions with complex terrain (Boyacá, COL7), where the temperature varied over smaller spatial scales, the increased resolution had a more pronounced impact on station-to-grid cell validations (see Table 3). For instance, BEST, re-gridded to a 0.5° grid, while not adding new spatial information, showed especially increased MAE and RMSE values in these areas, likely because it then better captured conditions near weather stations. Thus, there is evidence that increasing spatial resolution enhanced performance for lower-resolution data sets but slightly reduced it for higher-resolution ones, which still performed better. However, the general observation that ERA5 (0.25°) slightly outperformed ERA5Land (0.1°) in our station-to-grid cell analysis highlights the critical importance of data set accuracy over spatial resolution, which must be carefully considered.

### Translation into spatially aggregated area-level temperature estimates

Our findings regarding area-level temperature estimates aligned with our grid-based observational uncertainty analysis and showed that differences in GGTDs at the grid level translated into spatially aggregated averages. The spatial resolution of data sets must be particularly considered in remote areas and regions with complex terrain, even when evaluating temperature estimates spatially aggregated over extensive areas. The seasonality of the temperature and the size of the AD unit had little effect on the deviations of the data set, as shown by the Brazilian ValAr-P of varying seasonality and size, which did not consistently influence the differences of the GGTD in unit-specific temperature estimates.

These conclusions were drawn from our main results, which demonstrated that while the selection of GGTDs and their spatial resolution had minimal impact on aggregated time-series in most ValAr-P, higher-resolution data sets were generally preferable in remote and topographically complex regions. Similarly, for most ValAr-P, the comparison of timeseries derived from GGTDs on a common 0.5° grid or their native resolution exhibited negligible differences. However, in areas like the Andes-dominated mountainous region of Boyacá (COL7), spatially aggregated temperature timeseries exhibited greater variability. In COL7, where also the accuracy of both lower- and higher-resolution data sets varied with interpolation to a higher or lower grid, a stronger alignment of timeseries data was observed when comparing aggregated information derived from GGTDs on a common grid to those at their native resolution.

### Impact of observational uncertainty on simulated YF reproduction number

Our findings showed that data set choice can have substantial impacts on YF reproduction number estimates, particularly in regions with distinct characteristics, such as Amazonas (BRA4) in Brazil and the ValAr-P in Colombia (COL7 and COL20), where noticeable differences in spatially aggregated area-level temperature estimates were observed (see previous section). Notably, in Boyacá (COL7), pronounced variations in both the magnitude and temporal variations of the reproduction number became evident. While reproduction number values in Colombia remained well below 1.00, our results suggest that in regions where reproduction number approaches 1.00, data set differences could lead to substantial variations, with estimates shifting above or below the threshold depending on the selected GGTD. The shown critical sensitivity of the reproduction number to data input is not only important under current climate conditions but also in climate change impact studies assessing future outbreak potential due to human-to-human transmission, as we are confident that the underlying and observed mechanisms hold true for other regions with similar characteristics. To provide an example, a regional temperature increase based on GCM outputs downscaled using a simple delta change method might predict a higher reproduction number, potentially shifting from below 1.00 to above 1.00 in some areas when using BEST instead of ERA5Land, if ERA5Land provides cooler estimates, as observed in northern areas of South America.

Observational uncertainty is particularly critical in climate change impact studies when it matches or even exceeds uncertainties from other sources, such as variations among Representative Concentration Pathways (RCPs) and BC&D methods. Most important, it is especially relevant when variability across multiple observational data sets is comparable to or even surpasses that across GCMs. However, previous research suggests that observational uncertainty tends to be more significant for precipitation than for temperature in the context of future climate change projections [14,16,62,63]. For temperature, observational uncertainty is often smaller compared to uncertainties arising from the choice of BC&D methods, models, or scenarios. Nonetheless, we argue that observational uncertainty also in the context of temperature is neither negligible nor inconsequential in climate change studies due to the following reasons. First, it remains critical in applications such as the validation, ranking, and selection of RCMs and GCMs. Second, while prior research on VBD transmission and risk has often relied on oversimplified future climate change projections (see Introduction), future studies must integrate advanced BC&D methods that account for the entire distribution of climate variables. Reassessing and understanding the differences between data sets is essential, as we observed variations not only in mean responses but also in climate change-relevant extreme indices and bioclimatic variables, which current research has yet to fully explore in terms of their impact on the accuracy of both current and future VBD assessments. Lastly, in this context, the importance of observational uncertainty might vary depending on the specific research question or task at hand. For example, research on YF disease burden might prioritize emergency preparedness and worst-case scenarios. This could involve focusing on SSP585, a combination of the fossil-fueled development-based Shared Socioeconomic Pathway (SSP5) and the high-emission scenario RCP8.5, while also selecting extreme climate change scenarios based on disease-relevant indices or the tails of the temperature distribution. In such studies, areas where the reproduction number shifts, and whether future levels fall below or above the critical threshold of 1.00, could strongly depend on the choice of observational data source, leading to substantially different assessments of outbreak potential.

### Implications for practitioners and real-world decision-making

In practical terms, the challenge facing practitioners is no longer the scarcity of observational data or the availability of bias-corrected and downscaled climate projections. Rather, the critical issue lies in selecting an appropriate data set, rigorously assessing its credibility, and applying it judiciously. Often, data products are chosen based on availability, ease of use, or familiarity with the data provider. However, this approach can inadvertently introduce unrecognized biases into downstream analyses. To our knowledge, such biases have not been systematically evaluated in yellow fever (YF) SEIR modeling frameworks, which are increasingly used to inform policy and decision-making [34,35]. These models are increasingly adapted and extended to incorporate climate-related disease driving factors [64], underscoring the importance of critically assessing the choice and impact of input data to ensure robust and reliable simulations and projections of disease burden and transmission.

The underlying yellow fever (YF) modeling framework [10] used in this study integrates multiple further covariates, such as human population size, land cover, and non-human primate richness, all sourced from data sets with their own uncertainties. These covariates were held constant here to isolate and evaluate the impact of variability in temperature input data derived from GGTDs. Although a comprehensive uncertainty analysis would ideally extend to other environmental drivers, e.g., including the comparison of multiple land cover data sets beyond MODIS [65], such an approach was beyond the current scope, which explicitly focused on temperature-related data uncertainty and its influence on simulations of key YF epidemiological parameters. This focus was specifically intended to support the development and establishment of yellow fever modeling approaches that explicitly incorporate temperature sensitivity under current and future warming scenarios.

Looking ahead, accurately assessing the impacts of long-term climate change, including shifts in seasonality and occurrences of weather extremes, on disease projections and hence intervention and vaccination strategies depends critically on the choice or generation of appropriate climate change projection data sets. Advanced bias correction and downscaling techniques are indispensable for capturing seasonality and extremes, but their performance is highly sensitive to the reference data sets used, particularly for precipitation and extreme temperature indices [66]. Biases inherent in these reference data sets are transferred to the bias-adjusted and downscaled climate change projections, potentially amplifying errors in both current simulations and future projections when used in disease transmission models that are sensitive to driving data input. Raising awareness and utilizing the contributions identified in this study are essential steps toward promoting responsible and informed research practices. A careful examination of the data set documentation is crucial for researchers to understand its limitations, assumptions, and scope of application, guiding the decision on whether a new data source is needed and ultimately supporting the development of more accurate and reliable findings.

### Limitations

We note and discuss several limitations that may have influenced our findings. Multiple uncertainties exist regarding the grid-based evaluation, validation of GGTDs, and their spatial aggregation. Furthermore, we recognize limitations in our YF modeling approach, which, like any model, represents a simplification of real-world settings, with the current YF model having certain assumptions, detailed in [10]. In the following, we outline how some results may have been influenced by challenges in the study design, first concerning our station-to-grid cell analysis and subsequently with respect to our aggregation. We also discuss the limitations arising from the assumptions made with respect to the YF model.

### Station-to-grid cell analysis

Weather station data is often included in the generation of various GGTDs, such as reanalysis, although their inclusion is neither consistent over time nor uniform across all data sets. Any overlap between our selected stations and those used to develop the GGTDs could limit the independence and statistical validity of our analysis. In this context, we need to point out that our findings might have been generally influenced by temporal and spatial inconsistencies within the GGTDs, such as variations in the station data included over time in certain data sets. We acknowledge these inconsistencies could affect the reliability and accuracy of GGTDs, which are essential for long-term climate analysis and impact assessments.

Ground-based observations are limited to specific points in space and time, and data set performance might vary in locations where site-specific information was unavailable. Nonetheless, we argue that our focus on evaluating GGTD performance for health-related climate impact assessments, typically targeting inhabited regions, mitigated this issue. We leveraged the fact that weather stations in Brazil and Colombia were situated in or near densely populated areas, thereby limiting the impact of uneven station distribution on the validity of our results. In this context, it is important to note that both ERA5 and ERA5Land are known to underestimate temperature extremes in urban areas. This bias stems from the data assimilation schemes, which primarily rely on observations from official network stations. These stations are often not directly installed in urban locations, leading to a reliance on data from rural stations instead. This fact also needs to be considered when future research work evaluates the impact of temperature extremes on VBDs.

It is important to note that, in general, the values of the evaluation metrics indicated comparatively lower agreement between GGTDs and station data when compared to similar analyses conducted in high-income countries (similar findings have been reported in other studies, such as [12]), where station networks and data record-keeping are typically better maintained or have already been established over a longer period of time. In this context, we also highlight the general differences between the national station networks of IDEAM (Colombia) and INMET (Brazil), particularly with respect to the number of available stations, the lengths of historical records, and the ease of data accessibility. Although we acknowledge that both countries might differ in terms of infrastructure, historical development, and the institutional establishment of their meteorological services, we argue that global gridded data sets typically rely on national station networks, whether through direct use of station data for interpolation, blending with satellite-derived estimates, or integration via data assimilation techniques, as is the case with reanalysis products. As a result, structural disparities in national data infrastructure and meteorological networks are inherently reflected in the GGTDs themselves, representing a broader limitation when conducting cross-country analyses, particularly in the Global South. Despite applying consistent quality checks and data preparation procedures, we recognize that the limitations of weather station data must be taken into account when interpreting our findings. However, we argue that the differences between Brazil and Colombia do not substantially impact the overall conclusions of our study, since, for example, many of the structural discrepancies between the two countries are already embedded in the GGTDs. Our evaluation of GGTDs performance under local conditions revealed notable differences in accuracy, with stronger deviations observed in Colombia - likely more attributable to its complex topography in comparison with Brazil.

### Spatial aggregation

Spatial aggregation of GGTDs often involves averaging grid cells that may represent very different conditions within AD areas, influenced by factors such as size, orography, and climate. As a result, averaging these cells may not provide meaningful interpretations and could diminish the representativity and utility of the data. However, this method of spatial aggregation remains essential for certain applications, particularly in VBD research, which often relies on area-level epidemiological data that lack the spatial resolution needed to align with precise, more localized temperature information.

The impact of lakes and other water bodies was not evaluated, which is particularly relevant in regions with mixed land and water surfaces; while ERA5 includes data over lakes and water bodies, ERA5Land excludes them due to its land-sea mask, which considers only land areas. However, in most health-related climate research studies, gridded data sets are downloaded and spatially aggregated without further processing or corrections. Therefore, we also decided not to further account for respective differences.

### Modeling approach

The underlying YF modeling framework integrates multiple inputs, including static population data and a suite of climatic and environmental covariates. Following the approach detailed in prior work [67], a covariate selection process led to the exclusion of irrelevant variables, followed by clustering of remaining covariates and selection of the most representative from each cluster for stepwise model optimization based on the Bayesian Information Criterion. The current model therefore has certain assumptions, detailed in [10]. For example, while population data are incorporated, input remains static; dynamic processes such as migration, population growth, and additional socioeconomic factors, including poverty levels, are not represented. Furthermore, the distinct healthcare systems and organizational structures of Brazil and Colombia might influence their public health responses, potentially affecting the accuracy and completeness of disease and vaccination reporting. To better estimate population immunity, the YF modeling approach incorporates a measure of vaccination effectiveness that accounts for both vaccine efficacy and potential misclassifications or misreporting of vaccination coverage data. In general, like any model, the YF model represents a simplification of real-world conditions, with ongoing research needed to identify and, as knowledge and confidence improve over time, mechanistically incorporate additional driving factors where necessary.

## Conclusion

This study evaluated the uncertainty associated with observational global gridded temperature data sets, representing viable candidates for use as reference climatology for the bias correction and downscaling of global climate model simulations. The study focused on their impact on area-level temperature estimates and simulated yellow fever transmission. The study highlights the critical need to account for differences in data sets, which vary across contexts, time frames, and regions, and emphasize the sensitivity of yellow fever transmission to input data. Our findings underscore the need for careful evaluation and transparent reporting of observational uncertainties and the importance of selecting appropriate data sets to ensure robust climate (change) impact assessments in the context of disease transmission and outbreak potential. By providing evidence and foundational work to assess these uncertainties, this study offers guidance and raises awareness for policymakers, decision makers, and researchers, enabling them to make more informed decisions based on available and chosen data sources. Ultimately, improving the reliability of climate-informed disease risk assessments will enhance the design and implementation of effective intervention and vaccination strategies under current and changing climate conditions.

## Supplementary Material

Supplementary Material

## Figures and Tables

**Table 1:** Summary of the selected global gridded temperature data sets (GGTDs). All data sets were downloaded at their native resolution and with a (sub-) daily time resolution, except for Climatic Research Unit Time-Series data set (CRUTS), for which only monthly data were available. All data sets were accessed and extracted in 2023.

Product	Provider & Version	Native Resolution & Coverage	Further Details	Main Reference
BEST	Berkeley Earth Global Daily Land	1° × 1° Land-only	BEST reconstructs global mean land-surface temperatures using a mathematical framework that integrates around 39000 spatially and temporally divers weather station records of varying quality. The framework employs a weighting process to assess the quality and consistency of temperature station networks, enabling short, fragmented timeseries to be incorporated into the model.	[50]
CRUTS	University of East Anglia Climatic Research Unit v.4.07	0.5°× 0.5° Land-only	CRUTS is produced by interpolating monthly climate anomalies from a large network of weather stations using angular-distance weighting. Incorporating data from over 4000 weather stations, the data set is formatted consistently, though it is not entirely homogeneous despite efforts to homogenize many inputs.	[48]
ERA5	European Centre for Medium-Range Weather Forecasts	0.25° × 0.25°	ERA5 is generated using a four-dimensional variational data assimilation system, which combines model forecasts with observational data to produce gridded historical estimates of atmospheric conditions across the globe.	[47]
ERA5Land	European Centre for Medium-Range Weather Forecasts	0.1° × 0.1° Land-only	ERA5Land is an enhanced version of ERA5 that focuses on land surfaces, providing a higher resolution reprocessing of the land component. It uses ERA5 as input but with improvements in the land surface model to better capture processes specific to land areas.	[43]

**Table 2:** Definitions and calculations of temperature extremes, based on the Expert Team on Climate Change Detection and Indices (ETCCDI), and of bioclimatic variables (BCVs). ETCCDI calculations utilized daily maximum (TX) and minimum (TN) temperature timeseries; therefore, only data sets with daily resolution were selected (excluding Climatic Research Unit Time-Series data set, CRUTS). BCVs were calculated from monthly climatological averages and hence include all data sets. Indices and variables were computed using the global gridded temperature data sets at their native spatial resolutions as well as on a common 0.5° grid to account for uncertainties across different data sets.

Abbreviation	Full Name	Unit	Further Details
DTR	Daily temperature range	°C	Monthly mean difference between TX and TN. Here, the climatological monthly means averaged across the base period were considered. The DTR is exemplarily shown for January and July.
SU	Summer days index per time period	-	The number of summer days of a timeseries of daily TX. Days with TX > 25.00°C were counted.
TR	Tropical nights index per time period	-	The number of tropical nights of a timeseries of daily TN. Days with TN > 20.00°C were counted.
BCV1	Mean annual temperature	°C	
BCV2	Mean diurnal range	°C	Mean of maximum temperature – minimum temperature.
BCV3	Isothermality	-	(BCV2/BCV7) × 100.
BCV4	Temperature seasonality	-	Standard deviation × 100.
BCV5	Maximum temperature of warmest month	°C	
BCV6	Minimum temperature of coldest month	°C	
BCV7	Temperature annual range	°C	BCV5 - BCV6.
BCV10	Mean temperature of warmest quarter	°C	A quarter is defined as any consecutive 3 months.
BCV11	Mean temperature of coldest quarter	°C	A quarter is defined as any consecutive 3 months.

**Table 3:** Statistical evaluation of monthly temperature timeseries from grid cells against ground observations. The evaluation presents the Pearson correlation (PCC), the mean absolute error (MAE), and the root mean square error (RMSE) during the period 2011-2020 for each global gridded temperature data set (GGTD), averaged across each primary validation area (ValAr-P). Mean values represent averages across all validation areas (ValAr). Note that the number of weather stations evaluated varies across the different ValAr, as detailed in Tables S1 Table in the Supplement. Only stations with correlations statistically significant at the 95% confidence level were included in the analysis. Results are provided for all GGTDs at their native spatial resolution and on a common 0.5° grid (in brackets).

Country	Primary Validation Area (ValAr-P)	data set	PCC	MAE	RMSE
**Brazil**	Amazonas (BRA4)	BEST	0.67 (0.68)	0.82 (0.83)	0.94 (0.95)
		CRUTS	0.60 (0.61)	0.74 (0.74)	0.89 (0.89)
		ERA5Land	0.67 (0.67)	1.11 (1.14)	1.20(1.23)
		ERA5	0.69 (0.69)	0.85 (0.86)	0.96 (0.97)
	Rio de Janeiro (BRA19)	BEST	0.98 (0.97)	1.75 (1.81)	1.85 (1.93)
		CRUTS	0.98 (0.98)	1.91 (2.12)	2.00 (2.19)
		ERA5Land	0.98 (0.98)	2.02 (2.35)	2.10(2.42)
		ERA5	0.98 (0.98)	1.85 (1.88)	1.94(1.97)
	Rio Grande do Sul (BRA21)	BEST	0.99 (0.99)	1.59 (1.64)	1.74(1.80)
		CRUTS	0.99 (0.99)	1.51 (1.57)	1.66(1.72)
		ERA5Land	0.99 (0.99)	1.30 (1.30)	1.47(1.47)
		ERA5	0.99 (0.99)	1.36 (1.36)	1.52(1.52)
	Sergipe (BRA26)	BEST	0.95 (0.95)	1.13 (1.14)	1.24(1.24)
		CRUTS	0.95 (0.94)	1.13 (1.14)	1.26(1.28)
		ERA5Land	0.98 (0.97)	1.11 (1.10)	1.19(1.20)
		ERA5	0.97 (0.98)	1.10 (1.11)	1.18(1.18)
	**Mean**	**BEST**	**0.88 (0.88)**	**1.50 (1.50)**	**1.64 (1.64)**
		**CRUTS**	**0.86 (0.86)**	**1.46 (1.49)**	**1.60 (1.63)**
		**ERA5Land**	**0.88 (0.88)**	**1.42 (1.44)**	**1.57 (1.59)**
		**ERA5**	**0.89 (0.89)**	**1.36 (1.35)**	**1.49 (1.49)**
**Colombia**	Boyaca (COL7)	BEST	0.56 (0.58)	8.21 (6.36)	8.35 (6.57)
		CRUTS	0.68 (0.70)	5.86 (5.70)	6.11 (5.96)
		ERA5Land	0.61 (0.60)	3.95 (4.65)	4.50 (4.97)
		ERA5	0.66 (0.67)	4.44 (4.97)	4.78 (5.22)
	Magdalena (COL20)	BEST	0.77 (0.84)	0.58 (0.44)	0.72 (0.55)
		CRUTS	0.83 (0.83)	1.07 (1.30)	1.19(1.40)
		ERA5Land	0.80 (0.83)	2.79 (2.94)	2.86 (3.00)
		ERA5	0.84 (0.83)	1.80 (1.72)	1.87(1.79)
	**Mean**	**BEST**	**0.69 (0.70)**	**5.64 (5.29)**	**5.72 (5.38)**
		**CRUTS**	**0.69 (0.69)**	**4.88 (4.86)**	**4.98 (4.96)**
		**ERA5Land**	**0.72 (0.71)**	**4.91 (4.90)**	**5.01 (4.99)**
		**ERA5**	**0.74 (0.74)**	**4.74 (4.77)**	**4.83 (4.85)**

## Data Availability

All datasets utilized for the analysis and presented in this manuscript are publicly available. The ERA5 (representing the fifth generation ECMWF reanalysis) and ERA5Land (based on replaying the land component of the ECMWF ERA5 climate reanalysis) data sets were downloaded from the Copernicus Climate Change Service, available at https://cds.climate.copernicus.eu/. CRUTS (version 4.07) is available at [44] and BEST (Global Daily Land - Experimental; 1880 – Recent) is available at [46]. INMET station data were downloaded from [55], and IDEAM (Instituto de Hidrología, Meteorología y Estudios Ambientales, 2024) provided station data upon request (contacto@ideam.gov.co, enquiries received on 29-01-2024 and 09-02-2024). We downloaded spatial data of the GADM database (version 4.1) from GADM (2018-2022) at https://gadm.org/download_world.html, as well as extracted GPWv4 (revision 11) population data at [39]. Most of the data download, processing, and preparation were conducted using the R statistical software (version 4.3.2, IDE RStudio) and the Python programming language (Python 3.11.7, IDE PyCharm), as well as Climate Data Operators (CDO) [54], based on the provided scripting language package for Python (which is a wrapper around the CDO binary).
