# Supplementary material for: Evaluating the role of observational uncertainty in climate impact assessments: Temperature-driven yellow fever risk in South America
Source: PLOS Clim. Author manuscript; Available in PMC 2025 Dec 15. (PMC7618474; doi:10.1371/journal.pclm.0000601)
Supplement: Supplementary Material [file EMS211187-supplement-Supplementary_Material.zip › pclm.0000601.s001.pdf]

S1_Figures. Maps of validation areas (ValAr) and weather stations. A: Presentation of the selected ValAr in Brazil and Colombia, with secondary areas shown in pink and primary areas in green and additionally labeled. The locations of the weather stations used in this study are highlighted with red dots. B: An example of the spatial resolution differences across all global gridded temperature data sets (GGTDs), illustrated for one primary validation area (ValAr-P) in Brazil, Rio de Janeiro (BRA19). The temperature values represent the long-term annual climatological average, calculated by averaging monthly temperatures across all years within the base period (1991-2020).

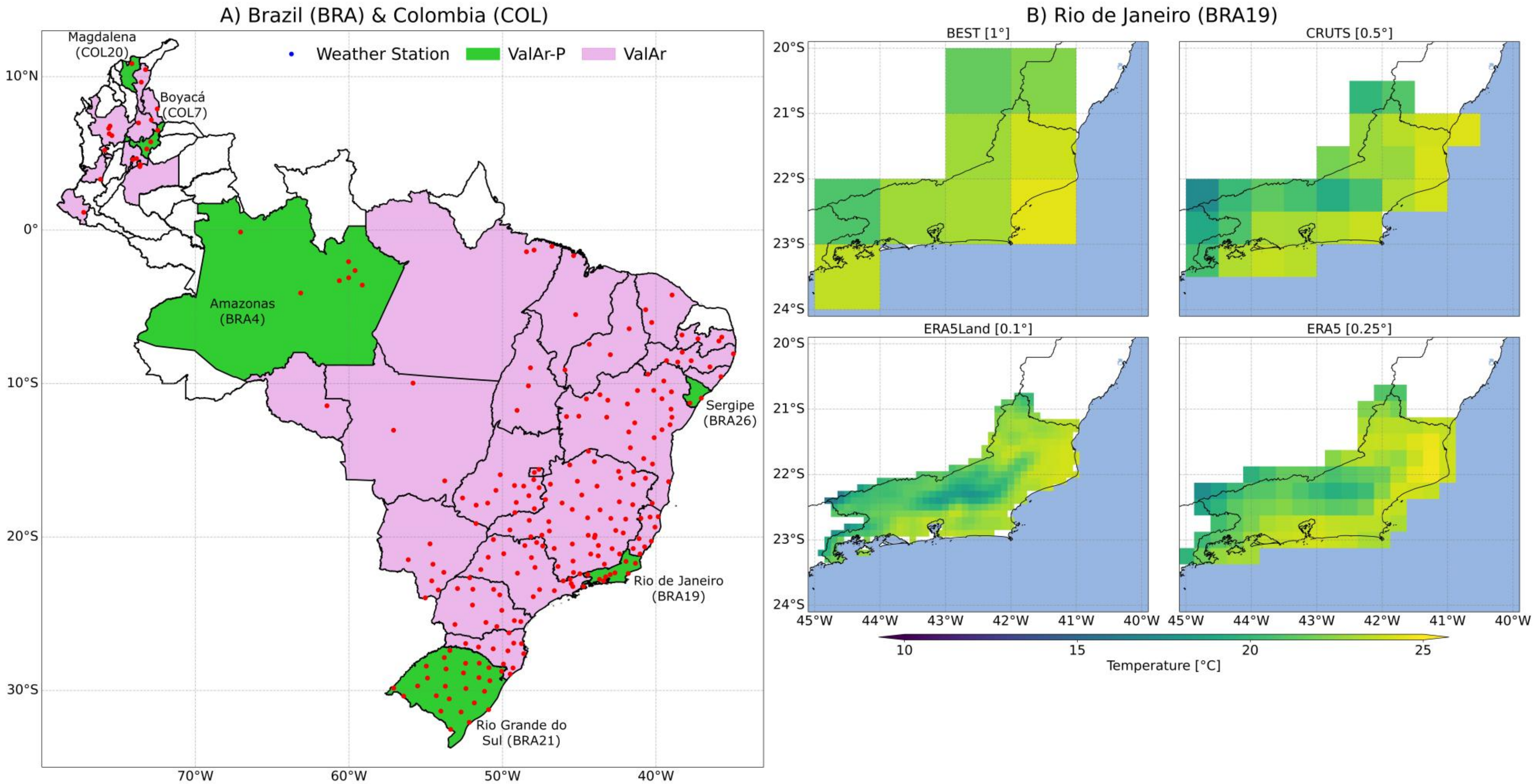
A) Brazil (BRA) & Colombia (COL)
Weather Station
ValAr-P
ValAr
Magdalena (COL20)
Boyacá (COL7)
Amazonas (BRA4)
Sergipe (BRA26)
Rio de Janeiro (BRA19)
Rio Grande do Sul (BRA21)
10°N
0°
10°S
20°S
30°S
70°W
60°W
50°W
40°W
B) Rio de Janeiro (BRA19)
BEST [1°]
CRUTS [0.5°]
ERA5Land [0.1°]
ERA5 [0.25°]
20°S
21°S
22°S
23°S
24°S
45°W
44°W
43°W
42°W
41°W
40°W
10
15
20
25
Temperature [°C]
