# Supplementary material for: Evaluating the role of observational uncertainty in climate impact assessments: Temperature-driven yellow fever risk in South America
Source: PLOS Clim. Author manuscript; Available in PMC 2025 Dec 15. (PMC7618474; doi:10.1371/journal.pclm.0000601)
Supplement: Supplementary Material [file EMS211187-supplement-Supplementary_Material.zip › pclm.0000601.s002.pdf]

S2_Figures. Differences in bioclimatic variables (BCVs) and metrics based on the Expert Team on Climate Change Detection and Indices (ETCCDI). The results are shown for further temperature-based BCVs (variables 2–5, 7, and 10–11) and indices defined by ETCCDI, including the daily temperature range (DTR) and the summer days index for specific time periods (SU). ERA5Land is used as the reference data set, and deviations are calculated for all other global gridded temperature data sets (GGTDs).

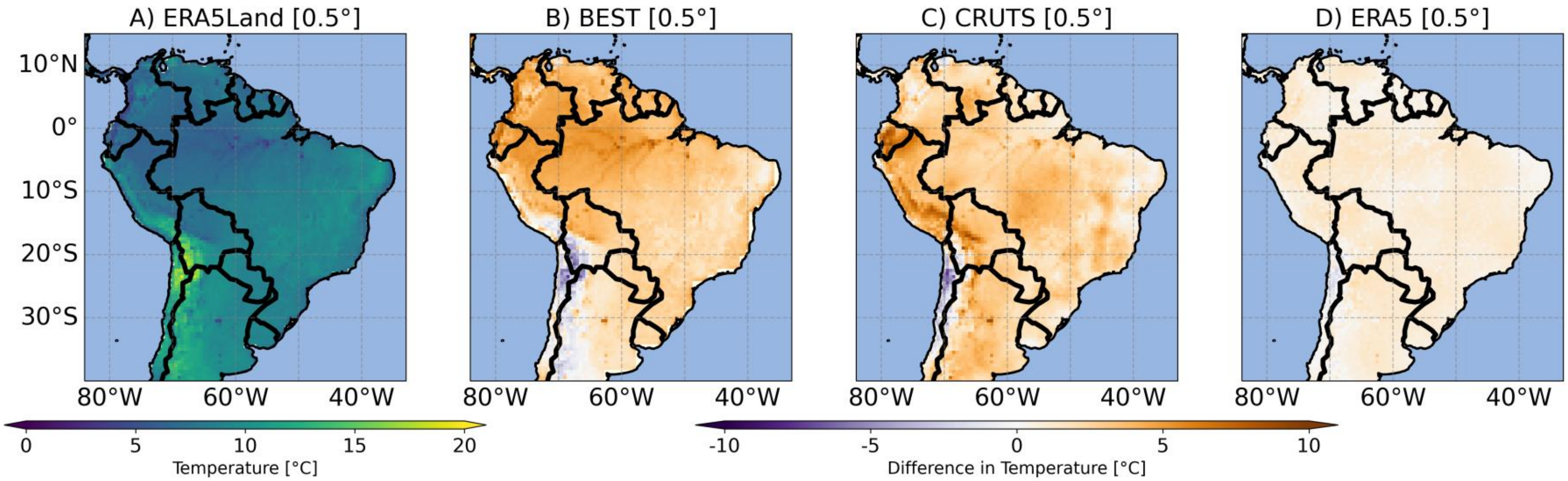

Fig S2. Bioclimatic variable 2 (BCV2). A: Mean diurnal temperature range [°C] for ERA5Land. B-D: For all other global gridded temperature data sets (GGTDs), the difference [°C] compared to ERA5Land is shown. All values represent averages over the base period (1991-2020). The maps are presented on a common 0.5° grid.

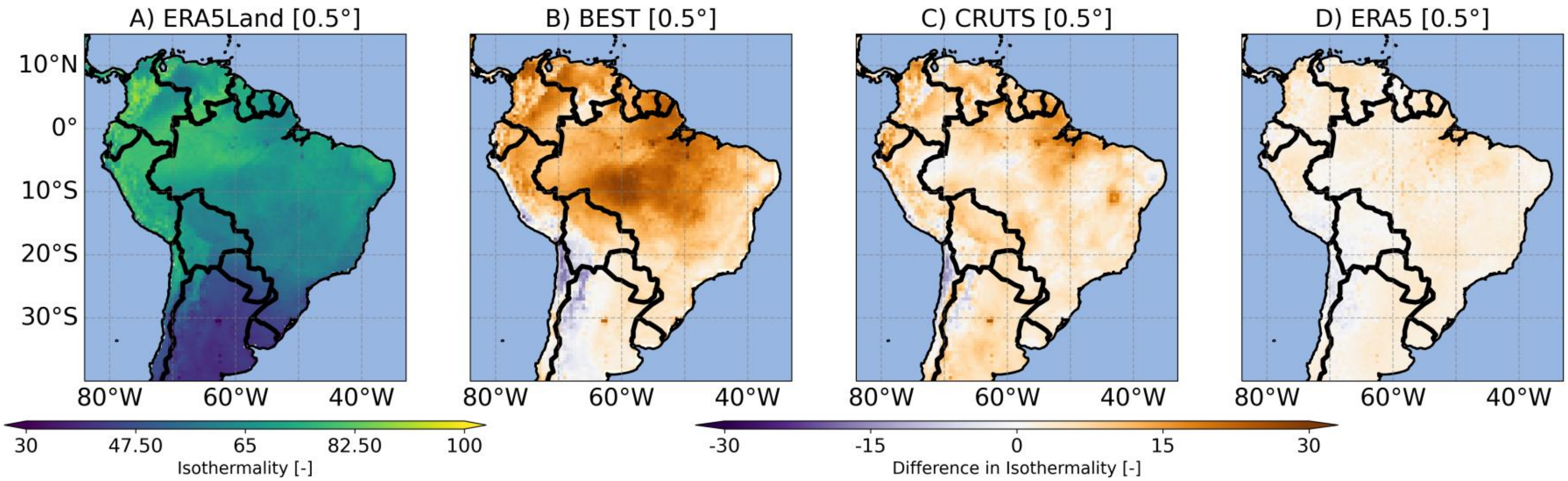

Fig S3. Bioclimatic variable 3 (BCV3). A: Isothermality [-] for ERA5Land. B-D: For all other global gridded temperature data sets (GGTDs), the difference [-] compared to ERA5Land is shown. All values represent averages over the base period (1991-2020). The maps are presented on a common 0.5° grid.

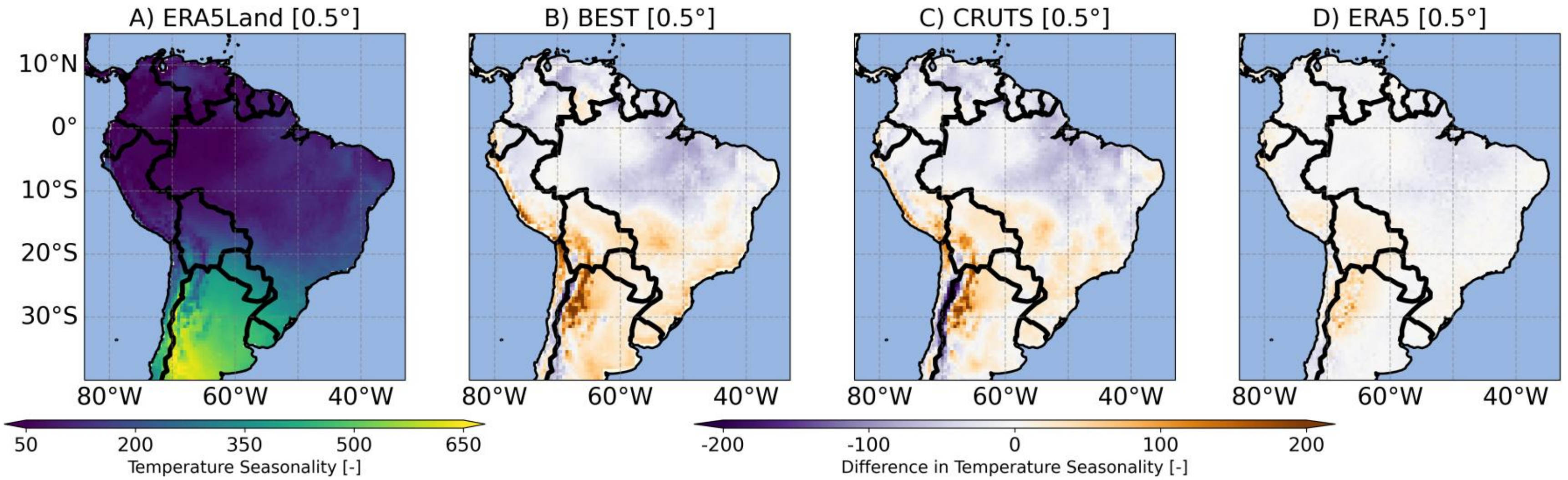

Fig S4. Bioclimatic variable 4 (BCV4). A: Temperature seasonality [-] for ERA5Land. B-D: For all other global gridded temperature data sets (GGTDs), the difference [-] compared to ERA5Land is shown. All values represent averages over the base period (1991-2020). The maps are presented on a common 0.5° grid.

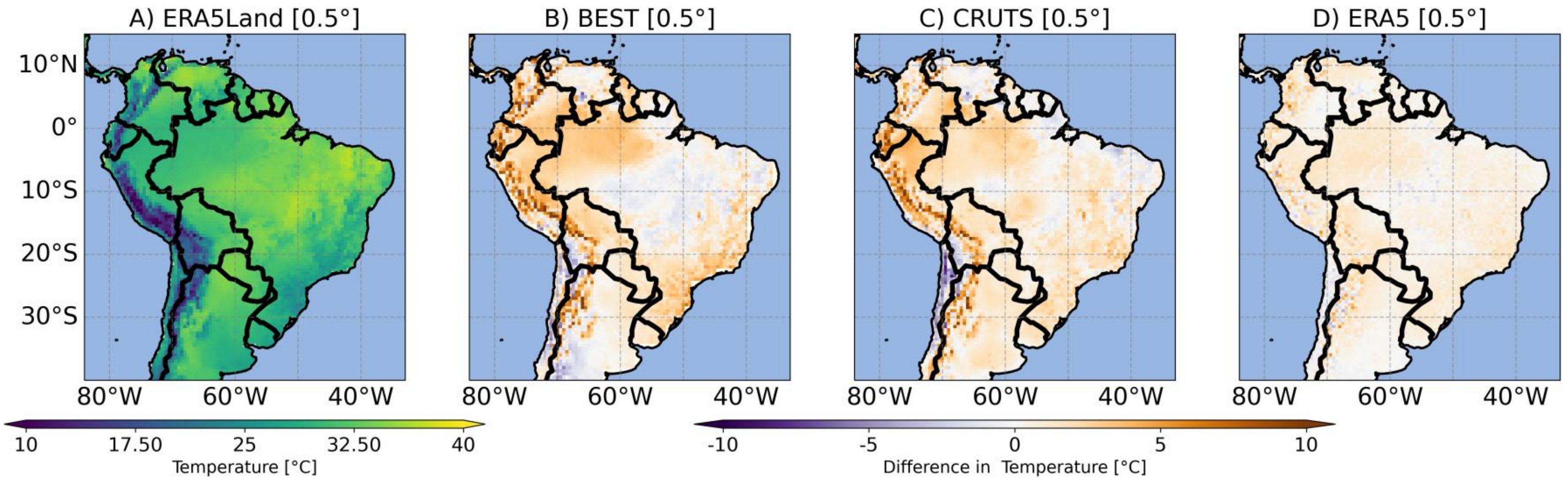

Fig S5. Bioclimatic variable 5 (BCV5). A: Maximum temperature of warmest month [°C] for ERA5Land. B-D: For all other global gridded temperature data sets (GGTDs), the difference [°C] compared to ERA5Land is shown. All values represent averages over the base period (1991-2020). The maps are presented on a common 0.5° grid.

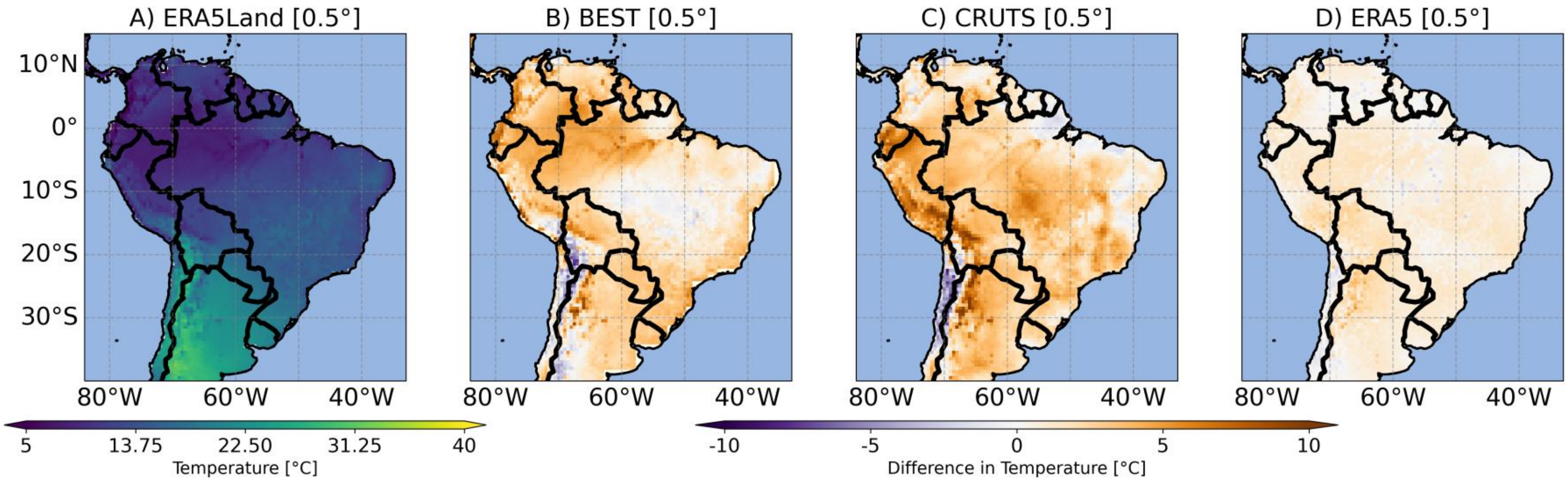

Fig S6. Bioclimatic variable 7 (BCV7). A: Temperature annual range [°C] for ERA5Land. B-D: For all other global gridded temperature data sets (GGTDs), the difference [°C] compared to ERA5Land is shown. All values represent averages over the base period (1991-2020). The maps are presented on a common 0.5° grid.

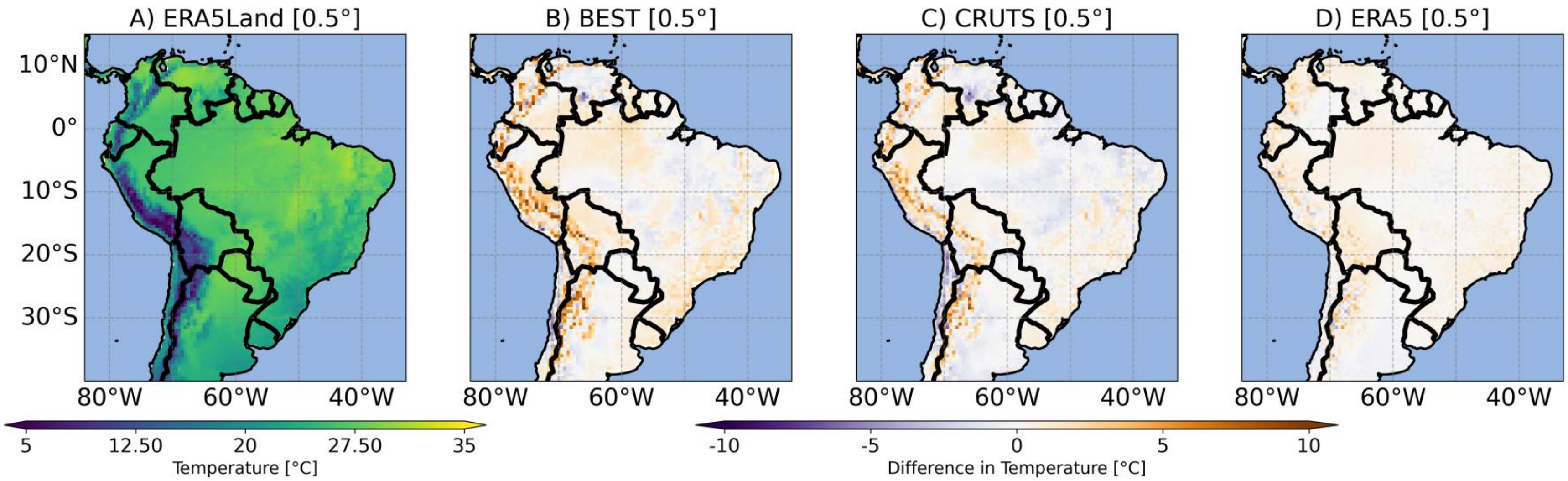

Fig S7. Bioclimatic variable 10 (BCV10). A: Mean temperature of warmest quarter [°C] for ERA5Land. B-D: For all other global gridded temperature data sets (GGTDs), the difference [°C] compared to ERA5Land is shown. All values represent averages over the base period (1991-2020). The maps are presented on a common 0.5° grid.

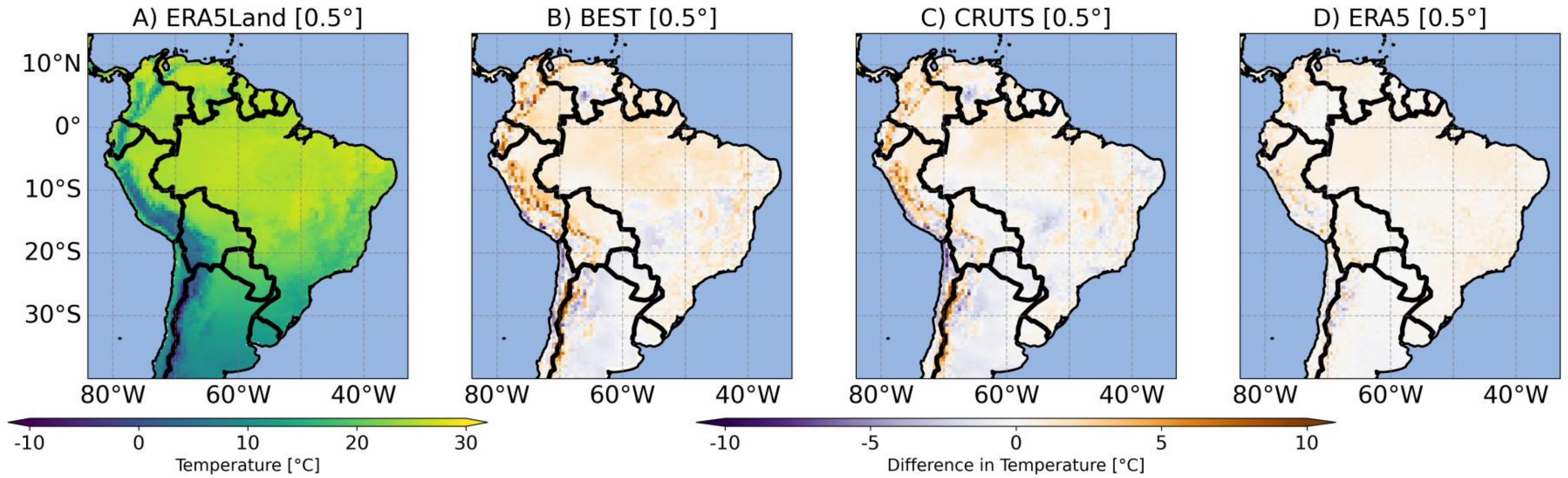

Fig S8. Bioclimatic variable 11 (BCV11). A: Mean temperature of coldest quarter [°C] for ERA5Land. B-D: For all other global gridded temperature data sets (GGTDs), the difference [°C] compared to ERA5Land is shown. All values represent averages over the base period (1991-2020). The maps are presented on a common 0.5° grid.

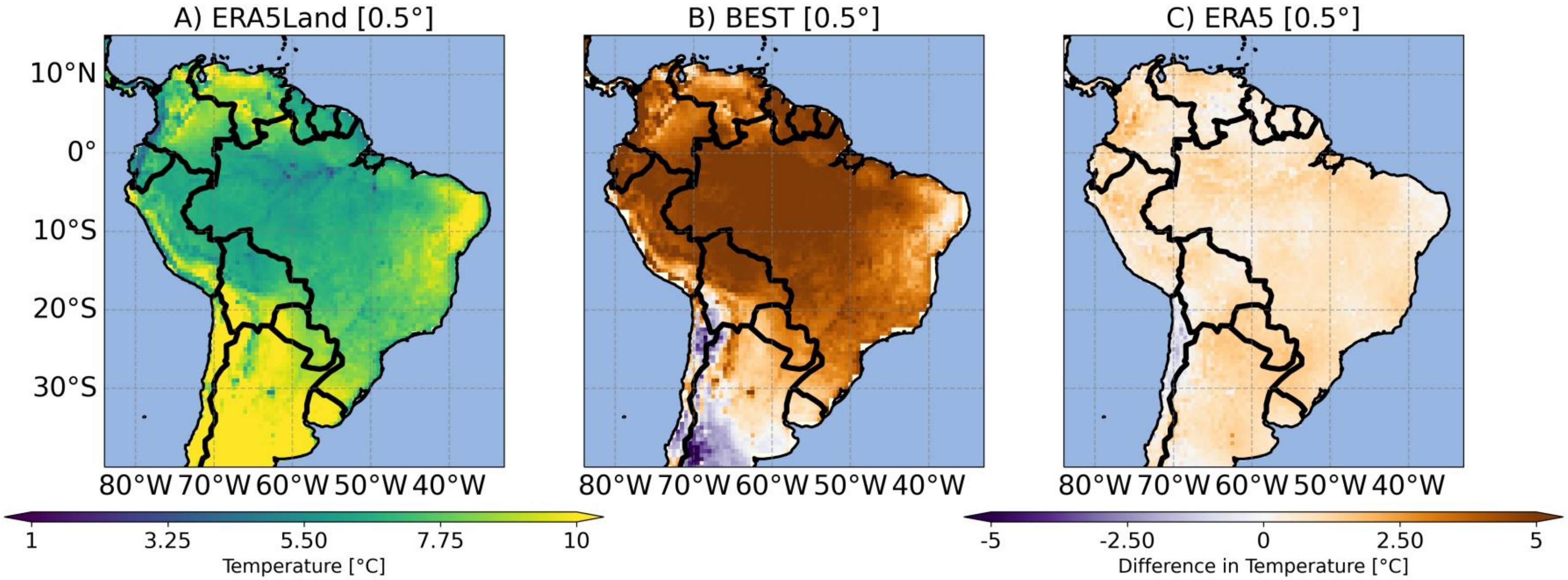

Fig S9. Daily Temperature Range (DTR) January. A: Daily temperature range [°C] for ERA5Land. B-C: For all other global gridded temperature data sets (GGTDs), the difference [°C] compared to ERA5Land is shown. All values represent averages over the base period (1991-2020). The maps are presented on a common 0.5° grid. Note that the Climatic Research Unit Time-Series (CRUTS) was excluded from the analysis, as DTR was calculated using daily values.

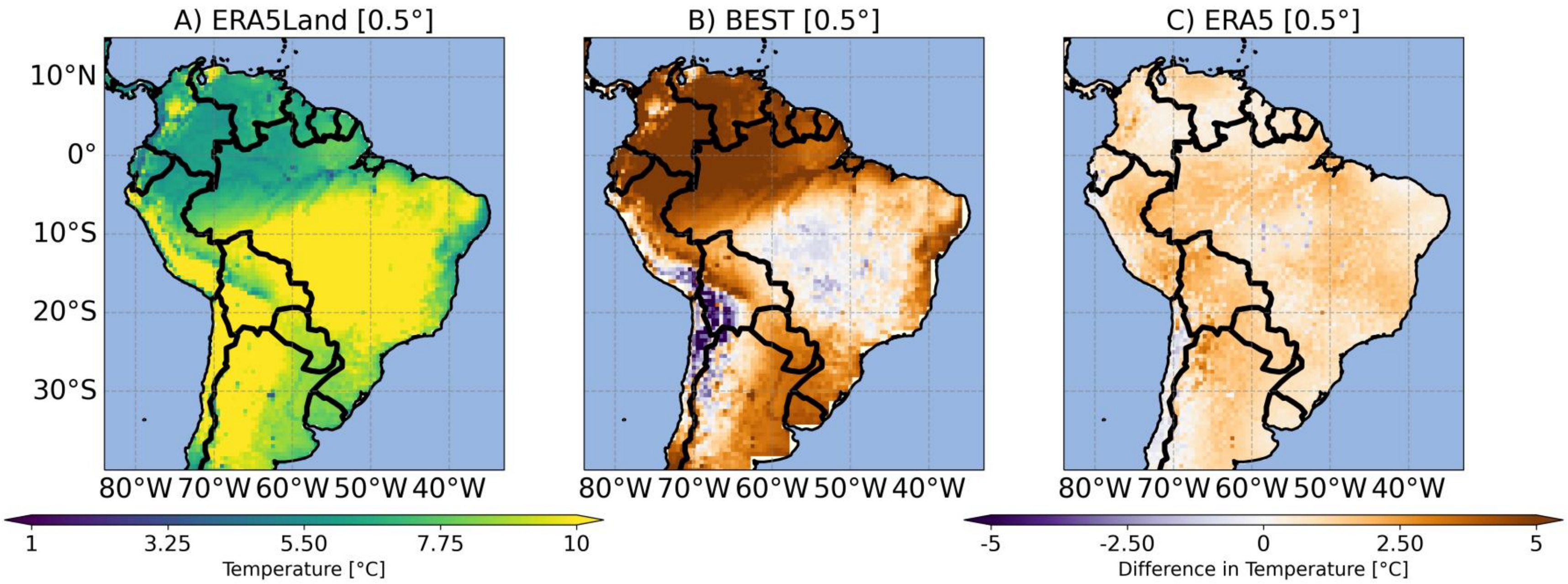

Fig S10. Daily Temperature Range (DTR) July. A: Daily temperature range [°C] for ERA5Land. B-C: For all other global gridded temperature data sets (GGTDs), the difference [°C] compared to ERA5Land is shown. All values represent averages over the base period (1991-2020). The maps are presented on a common 0.5° grid. Note that the Climatic Research Unit Time-Series (CRUTS) was excluded from the analysis, as DTR was calculated using daily values.

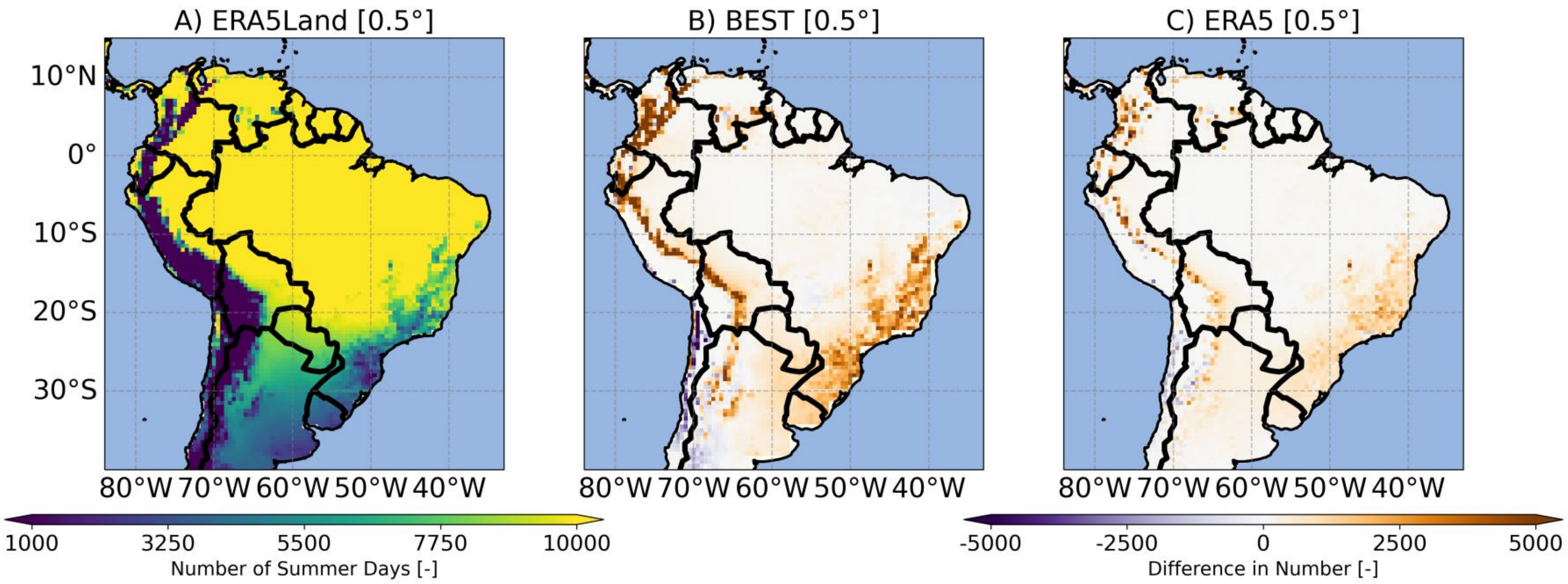

Fig S11. Summer days index per time period (SU). A: Number of summer days [-] for ERA5Land. B-C: For all other global gridded temperature data sets (GGTDs), the difference [-] compared to ERA5Land is shown. All values represent averages over the base period (1991-2020). The maps are presented on a common 0.5° grid. Note that the Climatic Research Unit Time-Series (CRUTS) was excluded from the analysis, as SU was calculated using daily values.
