# Supplementary material for: Evaluating the role of observational uncertainty in climate impact assessments: Temperature-driven yellow fever risk in South America
Source: PLOS Clim. Author manuscript; Available in PMC 2025 Dec 15. (PMC7618474; doi:10.1371/journal.pclm.0000601)
Supplement: Supplementary Material [file EMS211187-supplement-Supplementary_Material.zip › pclm.0000601.s003.pdf]

S3_Figures. Comparison of temperature distributions and climatological annual cycles across primary validation areas (ValAr-P) in Brazil and Colombia. The comparison is based on monthly time series from various global gridded temperature data sets (GGTDs). The validation areas include Amazonas (BRA4), Rio de Janeiro (BRA19), Rio Grande do Sul (BRA21), Sergipe (BRA26) in Brazil, and Boyacá (COL7) and Magdalena (COL20) in Colombia. All analyses are based on area-level time series derived from GGTDs on a common 0.5° grid.

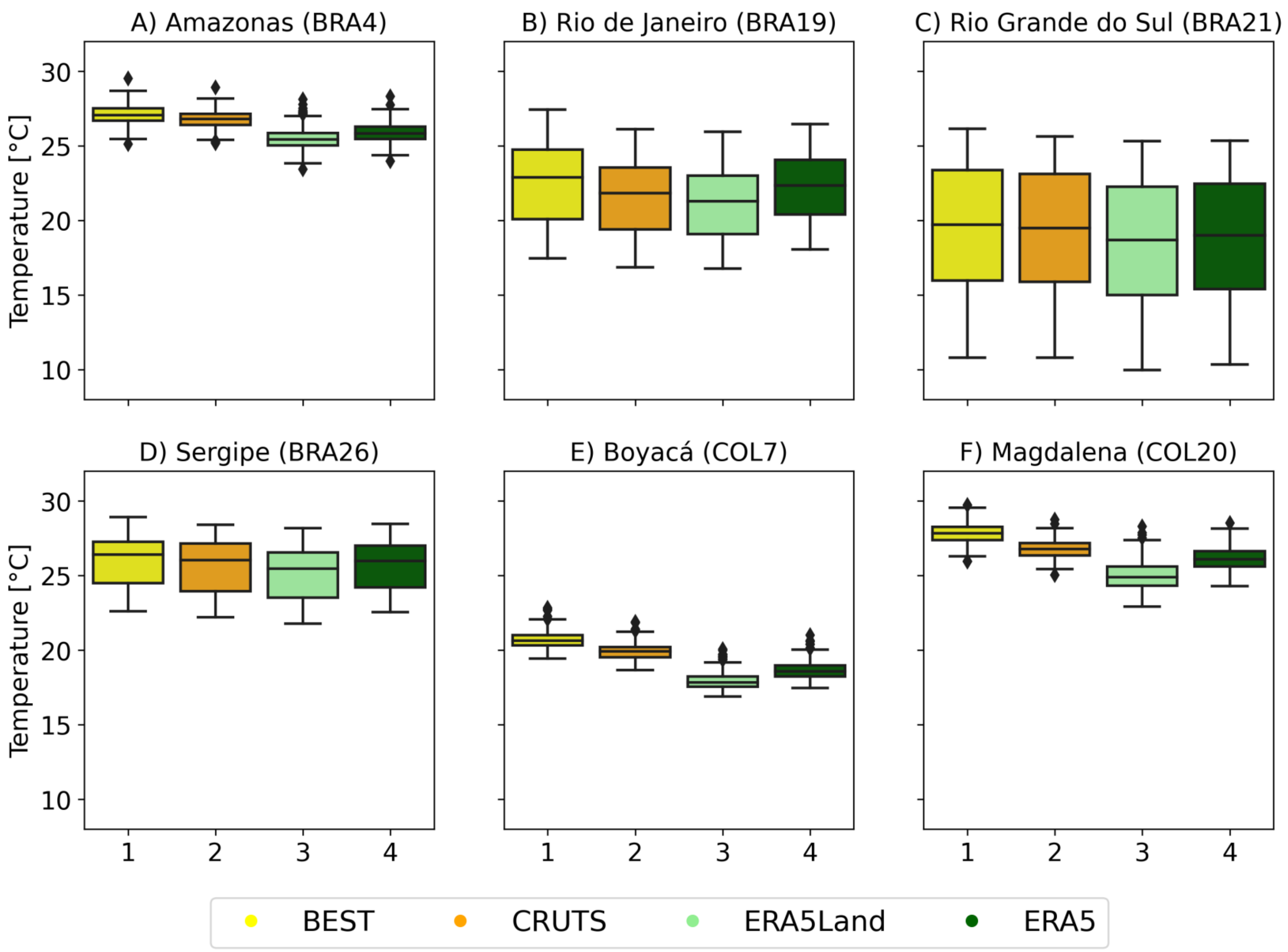

Fig S12. Boxplots comparing the temperature distributions of monthly timeseries based on different global gridded temperature data sets (GGTDs), averaged across each primary validation area (ValAr-P) in Brazil and Colombia, for the base period (1991-2020). The figures are organized as follows: A: Amazonas (BRA4) B: Rio de Janeiro (BRA19) C: Rio Grande do Sul (BRA21) D: Sergipe (BRA26) E: Boyacá (COL7) F: Magdalena (COL20) in Brazil (BRA) and Colombia (COL), respectively. These boxplots are based on area-level precipitation timeseries derived from GGTDs on a common 0.5° grid.

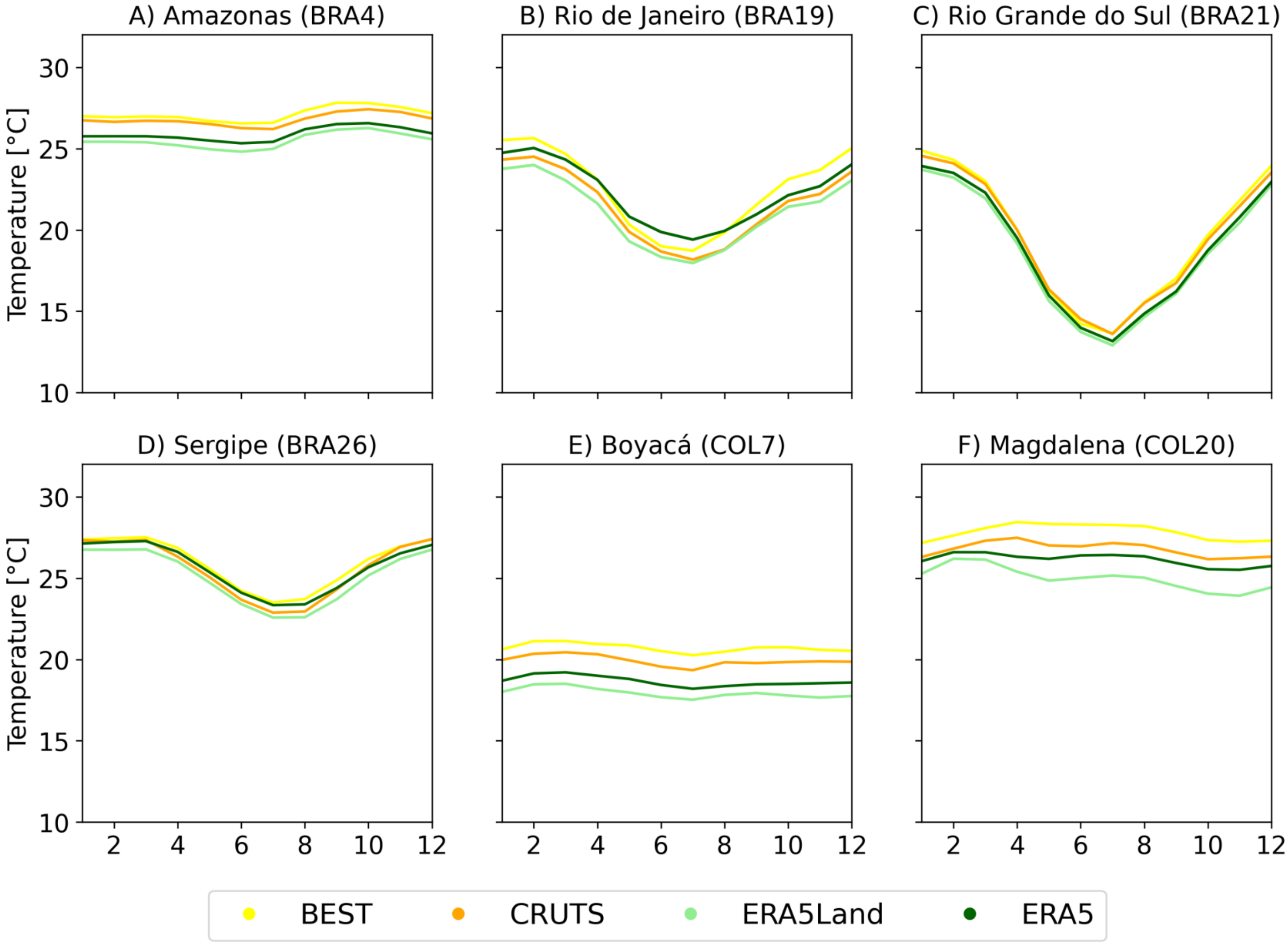

Fig S13. Climatological annual cycles of monthly temperature (°C) for the six primary validation areas (ValAr-P) selected across Brazil and Colombia, calculated over the base period (1991-2020). The figures are organized as follows: A: Amazonas (BRA4) B: Rio de Janeiro (BRA19) C: Rio Grande do Sul (BRA21) D: Sergipe (BRA26) E: Boyacá (COL7) F: Magdalena (COL20) in Brazil (BRA) and Colombia (COL), respectively. These climatological annual cycles are based on area-level precipitation timeseries derived from global gridded temperature data sets (GGTDs) on a common 0.5° grid.
