# Supplementary material for: Evaluating the role of observational uncertainty in climate impact assessments: Temperature-driven yellow fever risk in South America
Source: PLOS Clim. Author manuscript; Available in PMC 2025 Dec 15. (PMC7618474; doi:10.1371/journal.pclm.0000601)
Supplement: Supplementary Material [file EMS211187-supplement-Supplementary_Material.zip › pclm.0000601.s004.pdf]

S1_Glossary. Glossary. Overview of all in the paper used abbreviations and acronyms.

**Acronym / Abbreviation**	**Description**
AD (1)	(first-level) Administrative
BC & D	Bias correction & downscaling
BCV	Bioclimatic variable
BEST	Berkeley Earth Surface Temperatures data set
BRA	Brazil (country code)
CDO	Climate Data Operators
COL	Colombia (country code)
CRUTS	Climatic Research Unit Time-Series data set
DTR	Daily temperature range
ERA5 / ERA5Land	Reanalysis data set from the European Centre for Medium-Range Weather Forecast
ETCCDI	Expert Team on Climate Change Detection and Indices
FOI	Force of infection
GADM	Global Administrative Area Database
GCM	Global climate model
GGTD	Global gridded temperature data set
GPWv4	Gridded Population of the World, Version 4
HadCRUT5	Hadley Centre Climate Research Unit version 5 data set
IDEAM	Colombian Institute of Hydrology, Meteorology, and Environmental Studies
INMET	Brazilian National Institute of Meteorology

IPCC	Intergovernmental Panel on Climate Change
IQR	Interquartile range
LMIC	Low- and middle-income country
MAE	Mean absolute error
PCC	Pearson correlation coefficient
RCM	Regional climate model
RMSE	Root mean square error
SU	Summer days index per time period
TN	Daily minimum temperature
TR	Tropical nights index per time period
TX	Daily maximum temperature
ValAr(-P)	(Primary) Validation area
VBD	Vector-borne disease
W5E5 based on WFDE5	Merged data set based on the WATCH Forcing Data applied to ERA5
YF	Yellow fever
