# Supplementary material for: Evaluating the role of observational uncertainty in climate impact assessments: Temperature-driven yellow fever risk in South America
Source: PLOS Clim. Author manuscript; Available in PMC 2025 Dec 15. (PMC7618474; doi:10.1371/journal.pclm.0000601)
Supplement: Supplementary Material [file EMS211187-supplement-Supplementary_Material.zip › pclm.0000601.s005.pdf]

S1_Methods. Further details and equations on evaluation methods and metrics. The text provides a more detailed description of the three standard statistical methods, namely the Pearson correlation coefficient (PCC), the mean absolute error (MAE), and the root mean square error (RMSE), used to evaluate each individual global gridded temperature data set (GGTD) based on pixel-based values (P) against station observations (O), quantifying the degree of deviation from the reference (weather stations serving as ground truth).

$$PCC = \frac{\sum_{i=1}^{N}(P_i - \bar{P}) \times (O_i - \bar{O})}{\sqrt{\sum_{i=1}^{N}(P_i - \bar{P})^2} \times \sqrt{\sum_{i=1}^{N}(O_i - \bar{O})^2}}$$

$$MAE = \frac{\sum_{i=1}^{N} |O_i - P_i|}{N}$$

$$RMSE = \sqrt{\frac{\sum_{i=1}^{N}(O_i - P_i)^2}{N}}$$

The PCC ranges from -1 to +1, where a value close to +1 indicates a strong positive linear relationship between the respective GGTD and field-based station data; correlations are reported only when statistically significant at the 95% confidence level. Both the MAE and RMSE are well-established indicators of model and data set performance, quantifying discrepancies between GGTD outputs and observed ground-based station data. While MAE provides the average magnitude of errors in the same unit as the original data (C), RMSE gives more weight to larger discrepancies by squaring the differences between pixel-based and observed values before averaging.
