# Supplementary material for: Evaluating the role of observational uncertainty in climate impact assessments: Temperature-driven yellow fever risk in South America
Source: PLOS Clim. Author manuscript; Available in PMC 2025 Dec 15. (PMC7618474; doi:10.1371/journal.pclm.0000601)
Supplement: Supplementary Material [file EMS211187-supplement-Supplementary_Material.zip › pclm.0000601.s006.pdf]

S1_Table. Overview of the validation areas (ValAr) in Brazil and Colombia, with primary areas marked by an asterisk. The table provides additional details on the average population (pop) in 2010, area size (area), elevation (elev), and the number of pixels selected for spatial aggregation per global gridded temperature data set for each validation area, based on the respective gridded data sets (BEST, CRUTS, ERA5Land, ERA5). It also includes information on the number of weather stations (stat), the mean station elevation across all stations, and the elevation of the highest and lowest stations within each area. Additionally, descriptive statistics of temperature (mean, minimum and maximum values) for the evaluation period (2011-2020) are shown, based on timeseries data averaged across all stations within each ValAr.

ID	ValAr	Area [km²]	Elev [m]	Pop [total]	BEST	CRUTS	ERA5 Land	ERA5	Stat	Stat Elev [m]	Temperature [°C]
BRA.2	Alagoas	28608	210	3974797	8	20	267	61	1	84.12 (84.12, 84.12)	25.47 (23.14, 27.47)
BRA.4*	Amazonas	1570153	102	4529102	161	584	13112	2181	7	57.87 (19.9, 112.84)	26.7 (25.54, 29.04)
BRA.5	Bahia	592969	527	17445810	70	237	4907	858	23	501.65 (86.36, 1283.95)	24.44 (21.4, 26.84)
BRA.6	Ceará	150137	293	10230591	22	66	1283	238	3	524.86 (298.19, 865.53)	25.53 (23.4, 27.55)
BRA.7	Distrito Federal	6218	1034	3148591	4	6	61	15	2	1095.66 (1030.36, 1160.96)	21.43 (17.58, 25.08)
BRA.8	Espírito Santo	51876	313	4734364	10	30	437	92	8	120.31 (9.0, 507.48)	23.73 (20.01, 27.25)
BRA.9	Goiás	368516	630	7331033	46	151	3092	545	13	791.94 (491.17, 1211.08)	22.9 (17.09, 28.47)
BRA.10	Maranhão	332604	191	8007134	48	143	2849	515	3	157.91 (35.86, 283.69)	26.81 (25.04, 29.48)
BRA.11	Mato Grosso do Sul	406035	324	3072502	48	160	3273	566	8	386.09 (252.47, 528.47)	23.6 (17.67, 26.3)
BRA.12	Mato Grosso	952075	336	3666672	101	352	7815	1311	3	384.5 (293.71, 525.43)	25.6 (23.2, 28.53)
BRA.13	Minas Gerais	652280	734	24394410	76	252	5285	906	47	762.79 (189.11, 1544.89)	22.02 (17.34, 25.57)
BRA.14	Pará	1255417	196	9671640	129	464	10360	1760	3	24.63 (5.6, 47.13)	26.79 (25.19, 28.35)

BRA.15	Paraíba	57346	392	4624728	11	36	558	109	4	405.08 (237.04, 573.45)	25.36 (22.68, 27.41)
BRA.16	Paraná	241045	637	13537474	31	96	1895	332	12	647.04 (50.31, 1208.85)	20.47 (14.67, 25.19)
BRA.17	Pernambuco	100161	479	10813865	19	58	931	184	7	402.17 (11.3, 827.78)	25.85 (21.79, 28.96)
BRA.18	Piauí	256228	351	3770089	35	111	2235	396	3	360.97 (312.07, 398.83)	27.23 (24.47, 30.71)
BRA.19 *	Rio de Janeiro	50990	346	21123088	9	28	423	95	10	267.13 (3.0, 1070.0)	22.17 (17.81, 26.63)
BRA.21 *	Rio Grande do Sul	373701	273	14661123	42	134	2612	485	26	375.76 (3.82, 1228.59)	18.57 (11.35, 24.05)
BRA.22	Rondônia	246349	207	1867533	34	111	2111	373	1	183.53 (183.53, 183.53)	26.35 (24.64, 29.22)
BRA.24	Santa Catarina	120875	676	8664453	19	51	962	187	11	493.98 (2.0, 1400.06)	18.94 (11.1, 24.82)
BRA.25	São Paulo	289707	561	52768052	36	118	2327	427	16	692.42 (381.9, 1662.95)	21.6 (16.88, 25.59)
BRA.26 *	Sergipe	22701	159	2600959	8	16	214	44	2	104.26 (3.72, 204.8)	25.73 (23.12, 28.12)
BRA.27	Tocantins	286587	304	1715065	38	127	2486	437	3	243.57 (189.71, 274.0)	26.78 (24.9, 30.1)
COL.2	Antioquia	63857	976	7495734	16	40	603	119	4	1956.5 (1, 3221)	15.86 (14.81, 16.9)
COL.5	Bogotá D.C.	1644	3226	9263554	3	4	32	11	1	2646.0 (2646, 2646)	15.1 (14.17, 16.46)
COL.7*	Boyacá	23319	2252	1562959	11	25	262	57	3	2480.33 (1436, 3510)	14.57 (8.94, 16.68)
COL.12	Cesar	23004	492	1219101	8	19	243	50	2	122.0 (60, 184)	29.33 (26.94, 31.62)
COL.15	Cundinamarca	22518	1837	4098801	5	17	240	50	1	3205.0 (3205, 3205)	8.38 (3.01, 10.53)
COL.20 *	Magdalena	23507	423	1898413	8	16	225	50	1	30.0 (30, 30)	28.38 (26.35, 30.66)
COL.21	Meta	85676	423	1258859	14	44	777	150	2	567.0 (444, 690)	25.05 (22.65, 28.69)

COL.22	Nariño	31361	1137	2017470	8	20	290	65	1	2820.0 (2820, 2820)	12.98 (11.93, 14.26)
COL.23	Norte de Santander	22380	1233	1612529	7	17	232	52	1	311.0 (311, 311)	27.85 (24.6, 31)
COL.26	Risaralda	3566	1757	1176691	4	7	51	13	1	1748.0 (1748, 1748)	18.61 (17.01, 19.96)
COL.28	Santander	31010	1150	2592021	9	21	312	62	2	1715.0 (114, 3316)	18.3 (13.84, 19.99)
COL.31	Valle del Cauca	20990	1232	5436575	7	14	210	49	1	1615.0 (1615, 1615)	21.03 (19.46, 22.5)
